# NRF3 activates mTORC1 arginine-dependently for cancer cell viability

**DOI:** 10.1016/j.isci.2023.106045

**Published:** 2023-01-25

**Authors:** Shuuhei Hirose, Tsuyoshi Waku, Misato Tani, Haruka Masuda, Keiko Endo, Sanae Ashitani, Iori Aketa, Hina Kitano, Sota Nakada, Ayaka Wada, Atsushi Hatanaka, Tsuyoshi Osawa, Tomoyoshi Soga, Akira Kobayashi

**Affiliations:** 1 Laboratory for Genetic Code, Graduate School of Life and Medical Sciences, Doshisha University, 1**–**3 Miyakodani, Tatara, Kyotanabe, Kyoto 610–0394, Japan; 2 Laboratory for Genetic Code, Department of Medical Life Systems, Faculty of Life and Medical Sciences, Doshisha University, Kyotanabe, Kyoto 610–0394, Japan; 3 Institute for Advanced Biosciences, Keio University, Kakuganji, Tsuruoka 997-0052, Japan; 4 Division of Integrative Nutriomics and Oncology, RCAST, The University of Tokyo, 4-6-1 Komaba, Meguro-ku, Tokyo 153-8904, Japan; 5 Research Fellow of Japan Society for the Promotion of Science

**Keywords:** Cellular physiology, Cell biology, Cancer

## Abstract

Cancer cells coordinate the mTORC1 signals and the related metabolic pathways to robustly and rapidly grow in response to nutrient conditions. Although a CNC-family transcription factor NRF3 promotes cancer development, the biological relevance between NRF3 function and mTORC1 signals in cancer cells remains unknown. Hence, we showed that NRF3 contributes to cancer cell viability through mTORC1 activation in response to amino acids, particularly arginine. NRF3 induced *SLC38A9* and *RagC* expression for the arginine-dependent mTORC1 recruitment onto lysosomes, and it also enhanced RAB5-mediated bulk macropinocytosis and SLC7A1-mediated selective transport for arginine loading into lysosomes. Besides, the inhibition of the NRF3–mTORC1 axis impaired mitochondrial function, leading to cancer cell apoptosis. Consistently, the aberrant upregulation of the axis caused tumor growth and poor prognosis. In conclusion, this study sheds light on the unique function of NRF3 in arginine-dependent mTORC1 activation and the pathophysiological aspects of the NRF3–mTORC1 axis in cancer development.

## Highlights

• NRF3 transcriptionally induces arginine-dependent mTORC1 recruitment onto lysosome
• NRF3 enhances arginine loading via bulk macropinocytosis and selective transport
• NRF3-mTORC1 axis contributes to mitochondrial quality control and cell viability
• Aberrant upregulation of NRF3-mTORC1 axis causes tumor growth and poor prognosis

## Graphical abstract

**Figure:**
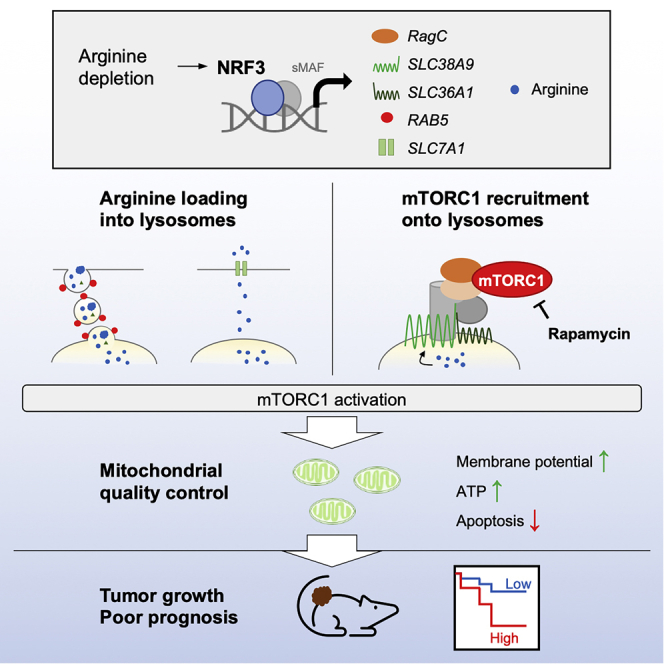

## Introduction

Cancer cells require large amounts of nutrients for rapid and continuous growth, and adapt to a low-nutritional environment through intracellular metabolic reprogramming.^1^ Specifically, the mechanistic (previously referred to as mammalian) target of rapamycin complex 1 (mTORC1) is the lysosomal membrane-localized serine/threonine kinase complex, which senses the nutritional conditions of cells and regulates cell proliferation.^2^ Of interest, a mTORC1 inhibitor, rapamycin, attenuates cancer development,^3^ implying the crucial role of mTORC1 in cancer metabolism reprogramming.

Nuclear factor erythroid 2-related factor 3 (NFE2L3, hereafter referred to as NRF3) belongs to the cap’n’collar (CNC) family of transcription factors.^4^ It is anchored to the endoplasmic reticulum (ER) membrane. Subsequently, it is processed by an aspartic protease, DNA damage-inducible 1 homolog 2 (DDI2), when the cell is subjected to stress conditions like proteasome inhibition.^5^ Furthermore, the processed NRF3 forms a heterodimer complex with a small MAF (sMAF) protein, such as MAFK, and then bind to an antioxidant response elements (ARE) on the target gene promoter, activating transcription.^4^ Therefore, NRF3 is a stress-inducible transcription factor, although its activation signals are hardly understood. Also, our previous studies showed the high expression of NRF3 genes in various cancer tissues and revealed that NRF3 promotes tumor growth through protein degradation of tumor suppressors, including p53 and retinoblastoma.^6^^,^^7^ Moreover, other groups reported the metastatic function of NRF3 in pancreatic and thyroid cancers.^8^^,^^9^ Recently, we observed that NRF3 activates sterol-responsive element-binding protein 2 (SREBP2), a master regulator of cholesterol biosynthesis. Then, it reprograms SREBP2-mediated lipid metabolism with cholesterol uptake through macropinocytosis, including bulk and fluid-phase endocytosis.^10^ Meanwhile, SREBP1, a master regulator of fatty acid metabolism, induces the gene expression of NRF1, the closest NRF3 homolog in the CNC family, through mTORC1 signals.^11^ These insights suggest that NRF3 is implicated in cancer metabolism and mTORC1 regulation, although their mechanisms remain unclear.

mTORC1 promotes anabolic pathways for the biosynthesis of building blocks, such as proteins, lipids, and nucleic acids, whereas it suppresses catabolic pathways, such as autophagy.^11^^,^^12^^,^^13^^,^^14^ For example, it promotes protein synthesis primarily through the phosphorylation of two key effectors: p70 ribosomal protein S6 kinase (S6K) and eukaryotic translation initiation factor 4E-binding protein 1 (4E-BP1).^15^ Also, mTORC1 promotes synthetic pathways, such as those of cholesterol biosynthesis and pentose-phosphate pathways, to supply lipids and nucleic acids required for cell growth.^13^^,^^16^ Moreover, mTORC1 further shifts glucose metabolism from oxidative phosphorylation to glycolysis for nutrient incorporation through mitochondrial quality control,^17^ whereas it prevents various catabolic processes, particularly autophagy, through the activation signal inhibition by AMP-activated protein kinase (AMPK).^18^ Thus, mTORC1 contributes to cell growth by balancing anabolism and catabolism.

mTORC1 activity is coordinated in response to environmental conditions by Rheb and Rag GTPase proteins, residing on the lysosomal surface.^19^^,^^20^^,^^21^^,^^22^ The activity of Rheb is regulated by a GTPase-activating protein (GAP), tuberous sclerosis complex (TSC). Meanwhile, Rag GTPase functions as a heterodimer, where RagA or RagB (RagA/B) binds to RagC or RagD, and the Rag complex is anchored to lysosomes via Ragulator, a pentameric scaffold protein complex.^23^ The Rag–Ragulator complex promotes the recruitment of mTORC1 onto the lysosomal membrane in response to several amino acids, such as leucine and arginine.^24^^,^^25^^,^^26^ Subsequently, growth factors, such as insulin, can promote the GTP binding of Rheb by inhibiting TSC and providing kinase activity to mTORC1. As described, the molecular basis for mTORC1 activation at protein levels has been increasingly elucidated. However, its transcriptional regulatory mechanism remains poorly understood.

This study showed that NRF3 is an arginine depletion-inducible transcription factor and it promotes tumor growth through arginine-dependent mTORC1 activation. Furthermore, we proved that NRF3 induces the recruitment of mTORC1 onto lysosomes by inducing the expression of *SLC38A9* and *RagC* genes in response to arginine levels. For mTORC1 activation, we also proposed that NRF3 enhances arginine loading into lysosomes through bulk macropinocytosis and selective transport. Metabolome and relevant functional analysis not only implicated NRF3 in glucose metabolism reprogramming but also indicated that the NRF3–mTORC1 axis contributes to mitochondrial function and cell viability. Consistently, aberrant upregulation of the NRF3–mTORC1 axis promoted tumor growth and predicted poor prognosis of several cancer types. In conclusion, these results provide a pathophysiological basis for establishing the NRF3 potential in cancer metabolism and development by arginine-dependently activating mTORC1.

## Results

### NRF3 contributes to mTORC1 activation

**Figure 1:**
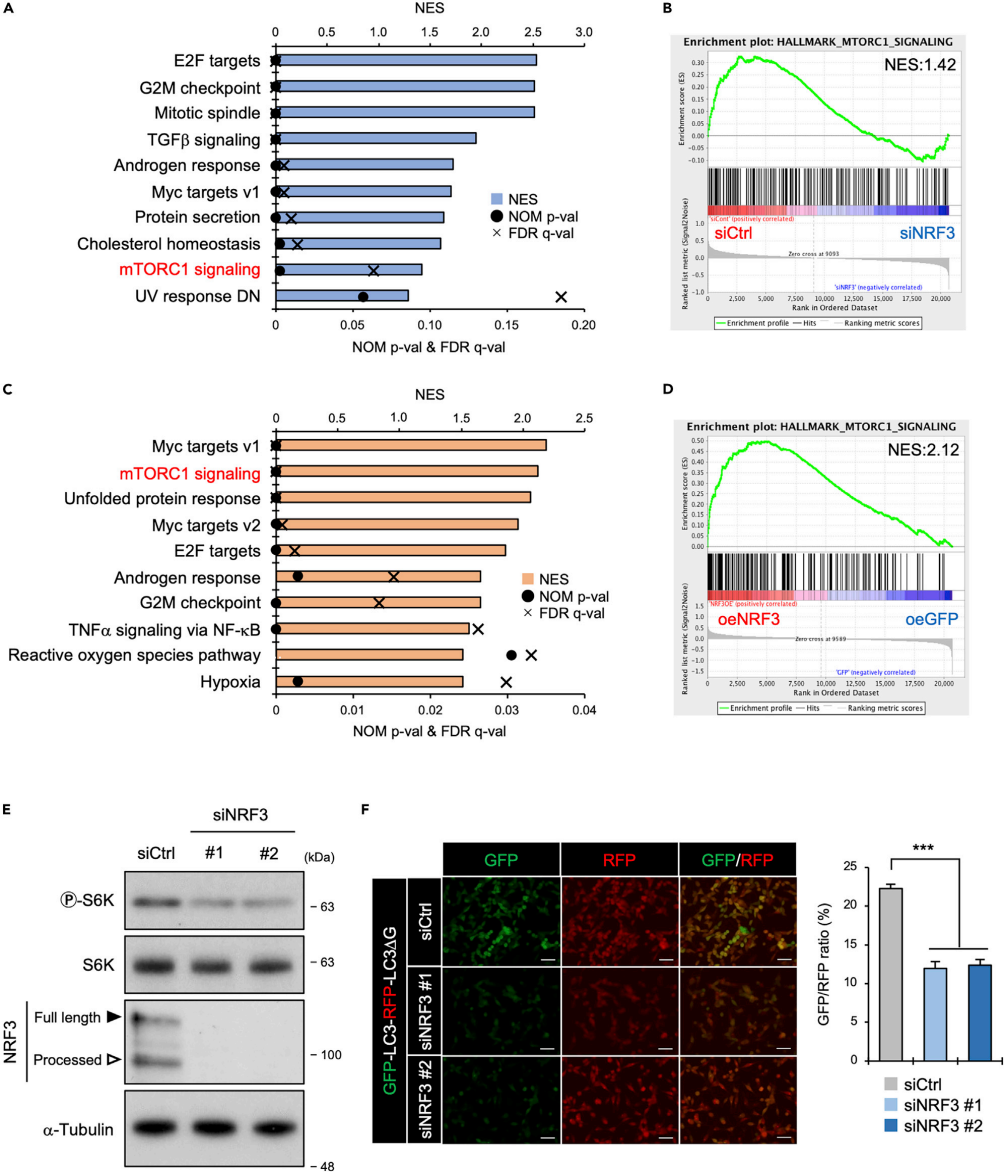
NRF3 is involved in mTORC1 activity (A–D) GSEA of HCT116-siNRF3 and siCtrl cells (A and B) or H1299-oeNRF3 and oeGFP cells (C and D). Previous DNA microarray datasets of HCT116 cells (GSE176444) or H1299 cells (GSE176444) were analyzed using open-source GSEA software v.3.0.^27^ In (A) and (C), the rank of gene sets is arranged by NES (normalized enrichment score) with NOM p-val (nominal pvalue) and FDR q-val (false discovery rate q-value). In (B) and (D), the enrichment plot of “mTORC1 signaling” is shown. (E) The effect of NRF3 knockdown on the basal activity of mTORC1. HCT116 cells were transfected with the indicated siRNA for one day. Control siRNA is represented as siCtrl. Full-length and processed NRF3 proteins are indicated with black and white arrowheads, respectively. (F) The effect of NRF3 knockdown on autophagy flux. HCT116 cells stably expressing the GFP-LC3-RFP-LC3ΔG probe were transfected with the indicated siRNA for two days. On the left panels, representative images are shown. Scale bars, 10 μm. In the right graph, GFP and RFP fluorescence intensities in the indicated cells were measured by flow cytometry, after which the GFP/RFP ratio was calculated. ANOVA followed by Tukey’s test: ∗∗∗p< 0.005 (n = 3, Mean ± SD). See also Figure S1.

Previously, we performed gene ontology analysis, adopting approaches that analyzed ranked gene lists filtered by a particular threshold, such as fold-change of the gene expression.^10^ Meanwhile, this study performed gene set enrichment analysis (GSEA), a threshold-free analysis for all genes, based on their differential expression rank, without prior gene filtering. GSEA results using our previous DNA microarray dataset of human colon cancer cells, HCT116-siRNA-mediated NRF3 knockdown (HCT116-siNRF3), and control (HCT116-siCtrl) showed that NRF3 expression was positively correlated with the “mTORC1 signaling” gene set (Figures 1A and 1B). Similar results were obtained using other human lung cancer cell datasets: H1299-NRF3 overexpression (H1299-oeNRF3) and control GFP overexpression (H1299-oeGFP) (Figures 1C and 1D). Subsequently, we investigated the impact of siNRF3 transfection on mTORC1 activation in HCT116, and showed that NRF3 knockdown reduced the phosphorylation levels of S6K (p-S6K), a marker of mTORC1 activation^15^ (Figure 1E). Also, we confirmed that the siNRF3 transfection did not change NRF3-related *NRF1* and *NRF2* mRNA levels (Figure S1A), and thus the above effects of NRF3 knockdown are not because of off-targeting on NRF1 and NRF2. In addition, NRF3 knockdown reduced p-S6K levels in other human cancer cell lines (colon cancer DLD-1 and pancreatic cancer PK-45H cells, including H1299 cells) where *NRF3* is endogenously expressed (Figure S1B). Followed by p-S6K, we further investigated whether NRF3 affects autophagy, which is suppressed by mTORC1 activation.^14^ As a result, NRF3 knockdown decreased GFP/RFP ratio from autophagy flux analysis using the GFP-LC3-RFP-LC3ΔG probe,^28^ indicating autophagy activation, thus, mTORC1 inactivation (Figure 1F). These results show that NRF3 is implicated in mTORC1 activation.

### NRF3 promotes the recruitment of mTOR onto lysosome in response to amino acid

**Figure 2:**
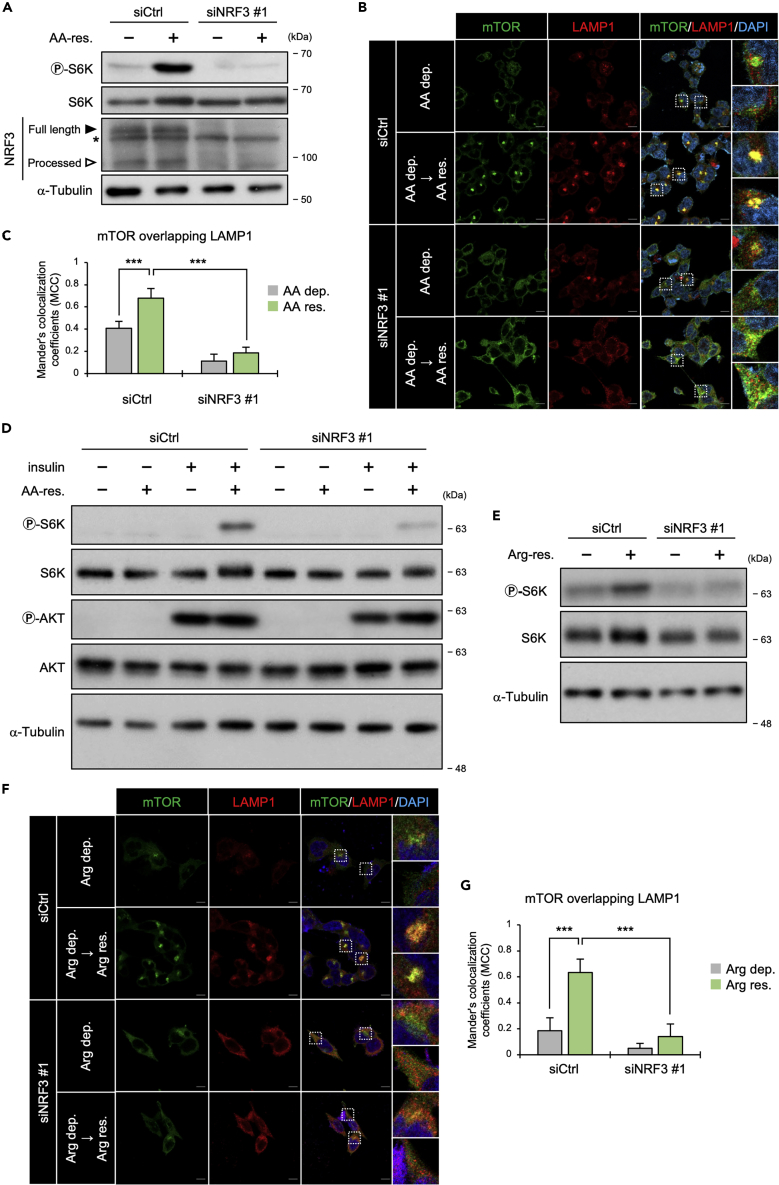
NRF3 arginine-dependently promotes the recruitment of mTOR onto lysosomes (A) The effect of NRF3 knockdown on mTORC1 activation in response to amino acid stimulation. For two days, HCT116 cells were transfected with the indicated siRNA. The cells were cultured without all amino acids for 5 h and then stimulated with or without all amino acids for 15 min (+/−AA-res.). Non-specific bands are shown as (∗). (B and C) The effect of NRF3 knockdown on mTOR recruitment onto lysosomes in response to amino acid stimulation. HCT116 cells were transfected and stimulated as in (A). The enclosed areas are enlarged on the right. Scale bars, 10 μm. In (C), Mander’s coefficient for mTOR overlapping LAMP1 (analyzing over 100 cells). (D) The effect of NRF3 knockdown on mTORC1 activation in response to amino acid and insulin stimulation. HCT116 cells were transfected with the indicated siRNA for one day. The cells were cultured without all amino acids and FBS for 5 h and then restimulated with all amino acids and/or 400 nM insulin for 30 min (+/−insulin). (E) The effect of NRF3 knockdown on mTORC1 activation in response to arginine stimulation. HCT116 cells were transfected with the indicated siRNA for one day. The cells were cultured without arginine for 5 h and then stimulated with arginine for 15 min (+/−Arg-res.). (F and G) The effect of NRF3 knockdown on mTOR recruitment onto lysosomes in response to arginine stimulation. HCT116 cells were transfected with the indicated siRNA for two days, Then, the cells were cultured without arginine for 5 h and then restimulated with arginine for 15 min. In (G), Mander’s coefficient for mTOR overlapping LAMP1 (analyzing over 100 cells). (C and G) ANOVA followed by Tukey’s test: ∗∗∗p< 0.005 (n = 3, Mean ± SD). See also Figure S1.

Amino acid restimulation following depletion (hereafter, the set of depletion and restimulation is referred to as the “stimulation”) induces mTORC1 activation. Then, we investigated whether NRF3 is essential for amino acid stimulation-mediated mTORC1 activation. The p-S6K levels were increased by amino acid stimulation, whereas the amino acid-dependent increase in p-S6K levels was abolished by NRF3 knockdown (Figure 2A). Furthermore, NRF3 knockdown reduced amino acid-dependent p-S6K levels in other cancer cells involving DLD-1, H1299, and PK-45H (Figure S1C). Amino acid stimulation controls mTORC1 localization to the lysosomal surface.^19^^,^^29^ Thus, we investigated whether NRF3 regulates the localization of mTORC1 on lysosomes by amino acid stimulation. First, we performed immunostaining using antibodies against two related proteins, including mTOR and lysosomal-associated membrane protein 1 (LAMP1) as a lysosome marker^30^^,^^31^ (Figures 2B and 2C); mTOR proteins were hardly immunocolocalized with LAMP1 proteins under amino acid depletion (AA-dep.). Besides, in response to the following amino acid restimulation, the immunocolocalization of mTOR proteins with LAMP1 proteins was enhanced (siCtrl in AA-dep.→AA-res.), although NRF3 knockdown impaired this immunocolocalization (siNRF3 in AA-dep.→AA-res.). These immunostaining results indicated that NRF3 promotes the mTOR recruitment onto lysosomes in response to amino acid stimulation, agreeing with the immunoblotting results of p-S6K above (Figure 2A). Recruited on the lysosome surface in response to amino acid, mTORC1 is then activated as a kinase by insulin-Rheb signaling. Thus we investigated the impact of insulin treatment on NRF3-mediated mTORC1 activation in response to amino acid. HCT116-siCtrl or siNRF3 cells were cultured in amino acid and serum free medium. In siCtrl cells, insulin was crucial for phosphorylation of S6K, similar to amino acid (Figure 2D, siCtrl). Meanwhile, NRF3 knockdown decreased phosphorylation of S6K in the presence of amino acid and insulin (Figure 2D, siNRF3). These results indicate that NRF3-mediated mTORC1 lysosomal recruitment affects mTORC1 activation by insulin treatment.

### Arginine is a crucial amino acid for NRF3-mediated mTORC1 activation

Then, we investigated which and how amino acid stimulation induces NRF3-mediated mTORC1 activation. Previous studies reported that leucine and arginine are also involved in mTORC1 activation.^15^^,^^32^ p-S6K levels were increased by both leucine and arginine (leucine/arginine) restimulation after leucine/arginine-depletion, although NRF3 knockdown abolished the leucine/arginine-dependent increase of p-S6K levels (Figure S1D), implying leucine and/or arginine is more crucial for NRF3-mediated mTORC1 activation. Leucine binds to Sestrin2, which dissociates it from GAP activity toward Rag 2 (GATOR2) complex, and activates mTORC1.^24^ In addition, glutamine acts upstream of leucine as an efflux solute to increase cytosolic leucine levels and activate mTORC1.^33^ Thus, we investigated the impacts of leucine and glutamine restimulation on the dissociation of Sestrin2 proteins from GATOR2 complex. To address this issue, we performed co-immunoprecipitation (Co-IP) experiment using an expression plasmid encoding FLAG-WDR24, a component of GATOR2 complex.^25^ Amino acid depletion increased Sestrin2 proteins co-immunoprecipitated with FLAG-WDR24 proteins and reduced the p-S6K levels, while the following amino acid restimulation reduced the co-immunoprecipitated Sestrin2 proteins and increased the p-S6K levels (Figure S1E, line 3 vs. 4). The similar results were obtained by the leucine and glutamine restimulation (Figure S1E, line 4 vs. 5). NRF3 knockdown did not affect the dissociation of Sestrin2 from FLAG-WDR24 in response to leucine and glutamine restimulation (Figure S1E, line 6 vs. 8), suggesting that leucine and glutamine are not likely to be key amino acids for NRF3-mediated mTORC1 activation. Then, we investigated whether arginine acts as a potential amino acid for NRF3-mediated mTORC1 activation, and found that NRF3 knockdown abolished the arginine-dependent increase of p-S6K levels (Figure 2E). More importantly, NRF3 knockdown also impaired the immunocolocalization of mTOR with LAMP1 in response to arginine stimulation (Figures 2F and 2G). These results indicated the possibility that NRF3 utilizes arginine to promote mTORC1 recruitment onto lysosomes. Moreover, this possible mechanism was further addressed in the next sections.

### NRF3 is activated by arginine depletion

**Figure 3:**
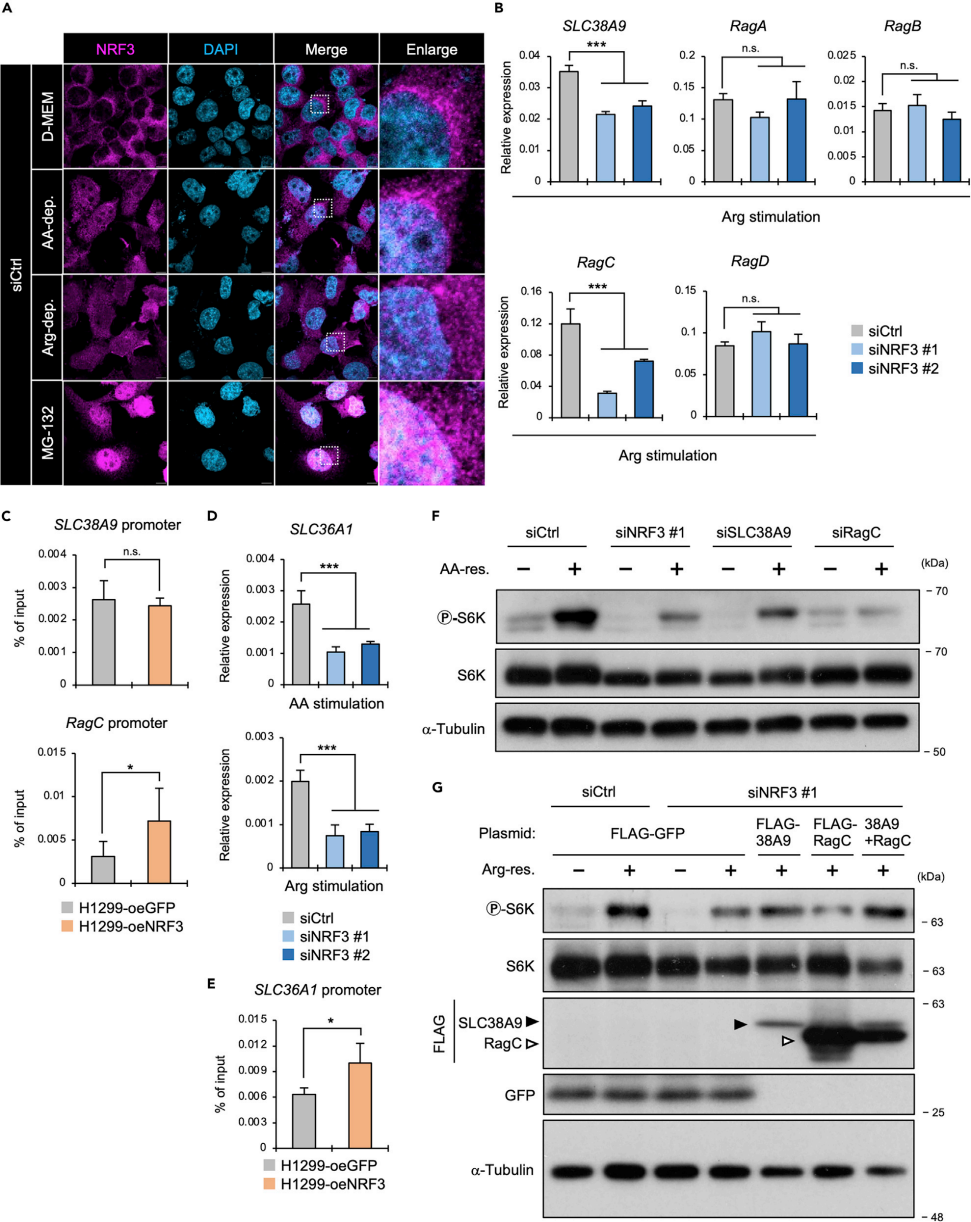
*SLC38A9* and *RagC* as crucial genes for NRF3-mediated arginine-dependent mTORC1 activation (A) The effect of amino acid or arginine depletion on the nuclear localization of NRF3. Related to Figure S2F, H1299-oeNRF3 cells were transfected with siCtrl for two days. For amino acid (AA) or arginine (Arg) depletion, the cells were cultured without all amino acids or arginine for 24 h (AA or Arg depletion). As a positive control, the cells were incubated with 1 μM MG-132 for 24 h. Scale bars, 10 μm. (B) The effect of NRF3 knockdown on the expression of five Ragulator-associated genes in response to arginine stimulation. For two days, HCT116 cells were transfected with the indicated siRNA. The cells were cultured without arginine for 5 h and then restimulated with arginine for 15 min. (C) The binding potential of NRF3 onto the *SLC38A9* or *RagC* promoter. H1299-oeNRF3 or oeGFP cells were treated with 1 μM MG-132 for 16 h. The ChIP-regions of each gene are shown in Figure S3B. (D) The effect of NRF3 knockdown on the expression of *SLC36A1* gene in response to amino acid (upper graph) or arginine (lower graph) stimulation. HCT116 cells were transfected and stimulated as in (B). (E) The binding potential of NRF3 onto the *SLC36A1* promoter. H1299-oeNRF3 or oeGFP cells were treated as in (C). The ChIP region is shown in Figure S3B. (F) The effects of the indicated gene knockdown on mTORC1 activation in response to amino acid stimulation. HCT116 cells were transfected and stimulated as in (B). (G) The add-back effect of *SLC38A9* and *RagC* genes on arginine-dependent mTORC1 activation in HCT116-siNRF3 cells. HCT116 cells were transfected with p3×FLAG-CMV 10 containing full-length (FLAG-GFP), pRK5 FLAG-SLC38A9.1 (FLAG-38A9, black arrowhead), and/or pRK5 FLAG-RagC (FLAG-RagC, white arrowhead). One day after plasmid transfection, the cells were transfected with the indicated siRNA for two days and then stimulated with arginine, as shown in (B). (C and E) Welch *t*-test, (B and D) ANOVA followed by Tukey’s test: ∗∗∗p< 0.005; ∗p< 0.05; n.s., not significant (n = 3, Mean ± SD). See also Figures S2 and S3.

NRF3 proteins are located in the ER membrane and subsequently are degraded by a proteasome. Once the cells undergo stress or stimulation, such as proteasome inhibition, using MG-132, NRF3 proteins are processed and then translocated to the nucleus for transcription activation.^5^ Unfortunately, we did not detect the processing and following nuclear translocation of endogenous NRF3 proteins in response to amino acid stimulation (data not shown). Thus, we used H1299-oeNRF3 cells in which the exogenous NRF3 proteins were processed and translocated to the nucleus in response to MG-132 treatment (Figures S2A and S2B, MG-132). Using this cell line, we then investigated the impact of amino acid stimulation on NRF3 protein processing in this study. Our results showed that amino acid stimulation increased processed NRF3 proteins (Figures S2A and S2B, AA stimulation). Subsequently, we subjected our findings to validation under arginine stimulation and observed similar results (Figures S2A and S2B, Arg stimulation). Furthermore, we investigated which stimulation treatment, including depletion and restimulation, is crucial for NRF3 activation, and found that amino acid or arginine depletion, but not the restimulation, leads to NRF3 protein processing (Figure S2C). Finally, we confirmed that amino acid or arginine depletion increased the nuclear translocalization of NRF3 proteins (Figure 3A), implying that amino acid, particular arginine depletion is a crucial cue for NRF3 activation, including the protein processing and following nuclear translocalization.

Then, we investigated the effect of amino acid or arginine depletion on DDI2, which is a crucial aspartyl protease for NRF3 protein processing.^5^ The DDI2 protein contains two conserved domains: ubiquitin-like (UBL) and retroviral protease-like (RVP) domains. Also, the catalytic site of DDI2 is formed through the homodimerization of an RVP domain.^34^ Of interest, Co-IP experiments using two expression plasmids encoding full-length DDI2 tagged with FLAG or HA showed that amino acid depletion increased the interaction between FLAG-DDI2 and HA-DDI2 (Figure S2D, Full). Similar results were obtained using UBL-deleted DDI2 (Figure S2D, ΔUBL). The bimolecular fluorescence complementation (BiFC) method using split Venus fragments also showed that the DDI2ΔUBL interaction was increased in response to amino acid depletion in living cells (Figure S2E). These results suggest that amino acid depletion trigger DDI2 homodimerization and subsequent NRF3 protein processing. Consistent with this suggestion, we found that DDI2 knockdown impaired the nuclear translocalization of NRF3 by amino acid or arginine depletion (Figure S2F), implying that NRF3 is activated in response to the depletion of amino acid, particular arginine through DDI2 homodimerization.

### NRF3 activates mTORC1 by inducing the expression of SLC38A9 and RagC in response to arginine stimulation

Next, we investigated the NRF3 target genes required for amino acid-dependent mTORC1 activation. Sestrin2 functions as a cytosolic leucine sensor, and SLC38A9 functions as a lysosomal arginine sensor for mTORC1 regulation.^26^ Also, SLC38A9 reportedly forms a complex with Ragulator and stimulates GDP release from RagA or RagB on its activation by arginine.^35^ RagA or Rag B is GTP-loaded, while other Rags, known as RagC or RagD, are GDP-loaded.^19^ This active conformation of the Rags promotes the recruitment of mTORC1 onto lysosomal surfaces. Hence, these insights imply that NRF3 uses the five above-mentioned Ragulator-associated genes: *SLC38A9*, *RagA, RagB, RagC, and RagD,* for arginine-dependent mTORC1 activation. Real-time quantitative PCR (RT-qPCR) results indicated that NRF3 knockdown significantly reduced the mRNA levels of *SLC38A9* and *RagC* under amino acid or arginine stimulation (Figures 3B and S3A). Also, *SLC38A9* and *RagC* promoters contained a conserved ARE within chromatin immunoprecipitation (ChIP) MAFK signals, which are heterodimer partners of NRF3 (Figure S3B). Furthermore, we performed ChIP-qPCR analysis using H1299-oeNRF3 or oeGFP cells. Results showed the possibility of NRF3 binding onto AREs regions in the *RagC* promoter but not the *SLC38A9* promoter (Figure 3C). A previous study reported that the SLC38A9 protein interacts with SLC36A1 proteins on the lysosomal surface, and these transporter complexes enhance each other’s expression levels.^36^ Expectedly, NRF3 knockdown reduced the gene expression of *SLC36A1* under amino acid or arginine stimulation (Figure 3D). Also, we observed that the *SLC36A1* promoter contained an ARE region, where NRF3 proteins were potentially recruited (Figures 3E and S3B). These results suggest that NRF3 directly induces *RagC* expression, whereas it indirectly induces *SLC38A9* expression through the direct gene expression of *SLC36A1*. Similar to NRF3 knockdown, *SLC38A9* or *RagC* knockdown also reduced p-S6K levels under amino acid stimulation (Figure 3F), implying that NRF3 activates mTORC1 through these gene expression in response to arginine stimulation. Subsequently, we added both *SLC38A9* and *RagC* genes back to HCT116-siNRF3 cells and observed the recovery of p-S6K levels reduced by NRF3 knockdown under arginine stimulation (Figure 3G). These results indicate that NRF3 induces the gene expression of *SLC38A9* and *RagC*, resulting in arginine-dependent mTORC1 activation. Moreover, NRF3 knockdown reduced the expression of *SLC38A9*, *RagC*, and *SLC36A1* at the mRNA and protein levels without any amino acid stimulation (Figures S3C–S3H). Therefore, based on the results that NRF3 knockdown reduces p-S6K levels without amino acid stimulation (Figure 1E), these results implicated NRF3 in the basal activity of mTORC1 in cancer cells through these gene expression.

### NRF3 enhances arginine loading through RAB5-mediated macropinocytosis and SLC7A1-mediated transport

**Figure 4:**
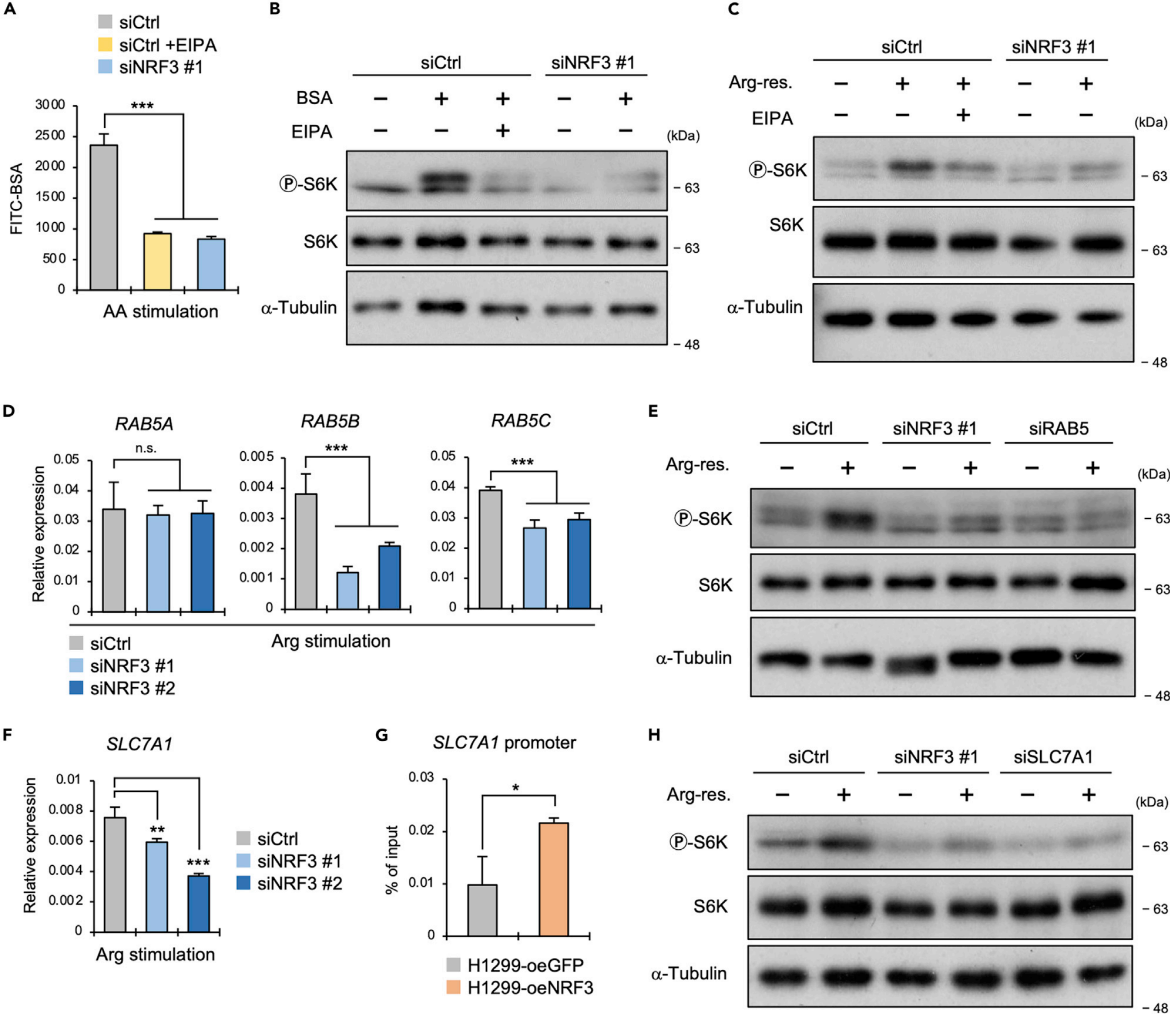
NRF3 induces RAB5-mediated macropinocytosis and SLC7A1-mediated amino acid transport for arginine-dependent mTORC1 activation (A) The effect of NRF3 knockdown on macropinocytosis induction in response to amino acid stimulation. HCT116 cells were transfected with the indicated siRNA and cultured with 1 μM EIPA for one day. Then, the cells were cultured without all amino acids for 5 h, after which they were restimulated with all amino acids and 1 mg/mL FITC-BSA for an additional 15 min. Median fluorescence intensity (MFI) values of FITC were measured by flow cytometry. As a control, HCT116-siCtrl cells were treated with 1 μM EIPA for one day and the stimulated amino acid and FITC-BSA. (B) The effect of BSA supplementation and/or macropinocytosis inhibition on mTORC1 activity. HCT116 cells were transfected with the indicated siRNA for one day. Then, the cells were cultured without both FBS and all amino acids for 5 h. Subsequently, these cells were stimulated with or without 5% BSA [w/v] for another 4 h (+/−BSA). As a control, HCT116-siCtrl cells were treated with 100 μM EIPA during BSA stimulation (+/−EIPA). (C) The effect of EIPA treatment on mTORC1 activation in response to arginine stimulation. HCT116 cells were transfected with the indicated siRNA for one day. Then, the cells were cultured without both FBS and arginine for 5 h. Finally, the cells were restimulated with or without arginine for 4 h (+/−Arg-res). As a control, HCT116-siCtrl cells were treated with 100 μM EIPA during arginine stimulation (+/−EIPA). (D) The effect of NRF3 knockdown on *RAB5s* expression in response to arginine stimulation. HCT116 cells were transfected with the indicated siRNA for two days. Then, the cells were cultured without arginine for 5 h. These cells were finally restimulated with arginine for another 15 min. (E) The effect of RAB5s knockdown on mTORC1 activation in response to arginine stimulation. HCT116 cells were transfected and cultured as in (C). siRAB5 means a mixture of siRAB5A, siRAB5B, and siRAB5C. (F) The effect of NRF3 knockdown on *SLC7A1* gene expression in response to arginine stimulation. HCT116 cells were transfected and cultured as in (D). (G) The binding potential of NRF3 to the *SLC7A1* promoter. H1299-oeNRF3 or oeGFP cells were treated with 1 μM MG-132 for 16 h. The ChIP region is shown in Figure S4H. (H) The effect of SLC7A1 knockdown on mTORC1 activation in response to arginine stimulation. HCT116 cells were transfected and cultured as in (D). (G) Welch *t*-test, (A, D, F) ANOVA followed by Tukey’s test: ∗∗∗p< 0.005; ∗∗p< 0.01; ∗p< 0.05; n.s., not significant (n = 3, Mean ± SD). See also Figure S4.

Macropinocytosis is crucial for mTORC1 activation through the bulk uptake of extracellular proteins and amino acids.^37^^,^^38^ Our recent study showed that NRF3 induces macropinocytosis and promotes the uptake of extracellular protein and lipid.^10^ Accordingly, we observed that NRF3 knockdown reduced the fluorescence intensity derived from a macropinocytosis indicator, fluorescein isothiocyanate-labeled bovine serum albumin (FITC-BSA) (Figure 4A), implying that NRF3 utilizes macropinocytosis for mTORC1 activation under amino acid stimulation. Next, to confirm this hypothesis, we investigated whether p-S6K levels were affected by BSA supplementation in a culture medium, following incubation with a macropinocytosis inhibitor, EIPA, and then knocking down NRF3. p-S6K levels were increased by BSA supplementation, while this enhanced effect of BSA supplementation was reduced by EIPA treatment (Figure 4B, siCtrl). This BSA-dependent increase in p-S6K levels was eliminated by NRF3 knockdown (Figure 4B, siNRF3). Similar results were obtained under amino acid or arginine stimulation (Figures 4C and S4A). Moreover, we found the consistent result that both BSA and the amino acid-dependent increase in p-S6K levels were further enhanced by NRF3 overexpression (Figures S4B and S4C). These results indicate that macropinocytosis induction is vital for NRF3-mediated amino acid-dependent mTORC1 activation.

Previously, we identified RAB5 genes, including *RAB5A*, *RAB5B*, and *RAB5C*, as NRF3-target regulators of macropinocytosis.^10^ As expected, *RAB5* gene expression levels were reduced by NRF3 knockdown under amino acid or arginine stimulation (Figures 4D and S4D). Also, we confirmed that RAB5 knockdown reduced the BSA- or arginine-dependent increase in p-S6K levels using siRAB5 oligonucleotides (Figures 4E and S4F), and NRF3 thus induces RAB5-mediated macropinocytosis for arginine uptake and the subsequent mTORC1 activation. In addition, NRF3 induced the gene expression of *SLC7A1*, a cationic amino acid transporter involving arginine, lysine, and ornithine^39^ (Figures 4F and S4G). Moreover, ChIP-qPCR results showed the binding potential of NRF3 to the ARE region in the *SLC7A1* promoter (Figures 4G and S4H), implying the functional role of *SLC7A1* for NRF3-mediated mTORC1 activation in response to amino acid stimulation. Therefore, to validate this point, we used a siSLC7A1 oligonucleotide (Figure S4E). Results confirmed that, similar to NRF3 knockdown, SLC7A1 knockdown reduced p-S6K levels increased by the stimulation of amino acids or arginine (Figures 4H and S4I). Hence, these results suggest that NRF3 induces RAB5-mediated bulk macropinocytosis and SLC7A1-mediated selective transport, enhancing arginine loading into lysosomes for mTORC1 activation.

### Inhibition of the NRF3–mTORC1 axis impairs mitochondrial function and cell viability

**Figure 5:**
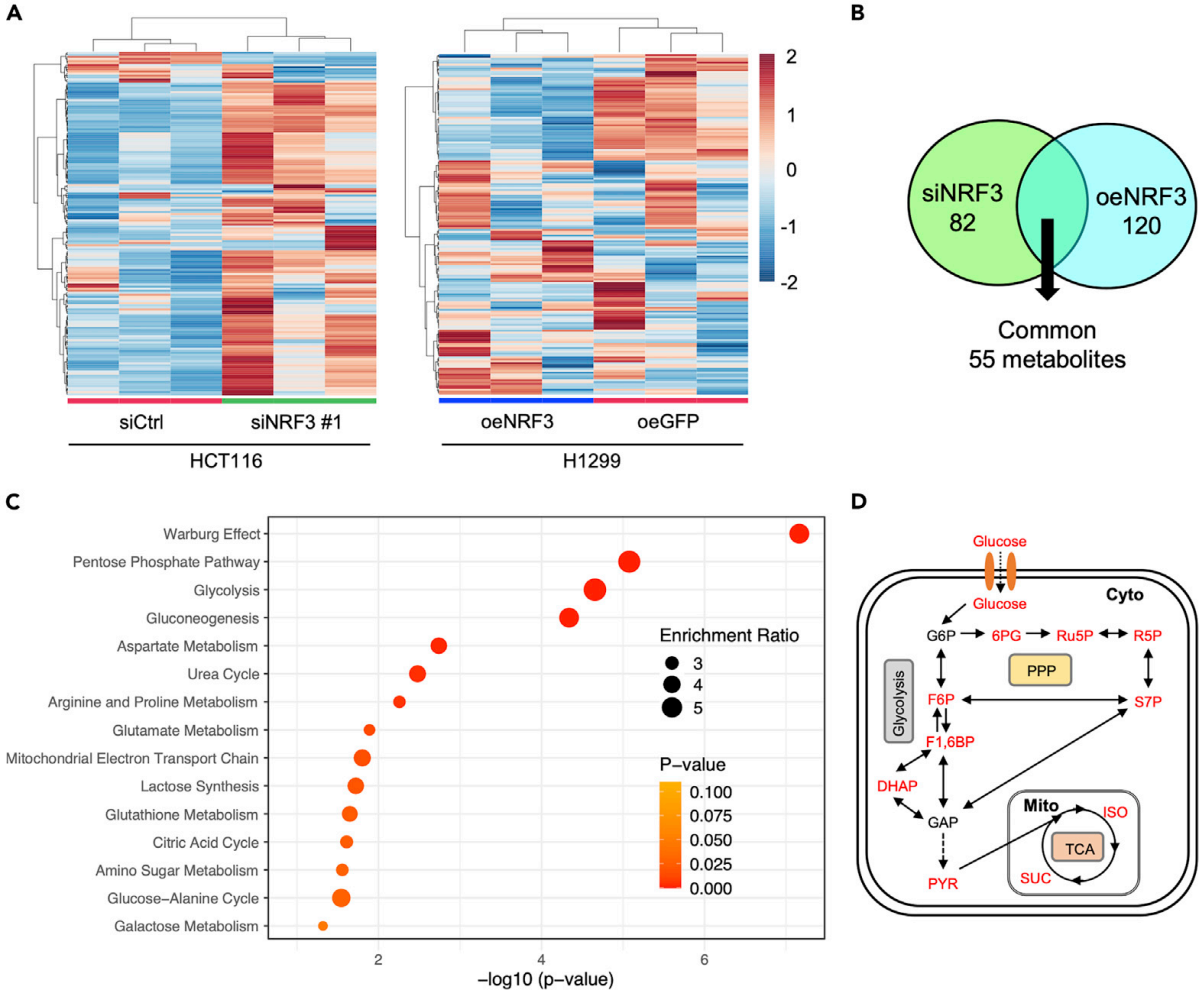
NRF3 is implicated in glucose metabolic pathways related to the Warburg effect (A) K-means clustering results of the metabolomics dataset derived from HCT116-siNRF3 and siCtrl cells with amino acid stimulation (left) or H1299-oeNRF3 and oeGFP cells without amino acid stimulation (right). (B) Venn diagram showing the up-or down-regulated metabolite sets in HCT116-siNRF3 cells (82 metabolites) and H1299-oeNRF3 cells (120 metabolites). (C) Enrichment analysis showing 55 common metabolites selected in (B). (D) A schematic representation showing NRF3-affected metabolite and metabolic pathways. Metabolites in the “Warburg effect” index shown in (C) are colored red. G6P, Glucose 6-phosphate; F6P, Fructose 6-phosphate; F1,6BP, Fructose 1,6-bisphosphate; DHAP, Dihydroxyacetone phosphate; GAP, D-glyceraldehyde-3-phosphate; PYR, Pyruvic acid; SUC, Succinic acid; ISO, Isocitric acid; 6 PG, 6-Phosphogluconic acid; Ru5P, D-Ribulose 5-phosphate; R5P, D-Ribose 5-phosphate; S7P, D-Sedoheptulose 7-phosphate. See also Figure S5, Tables S1, S2, S3, and S4.

mTORC1 controls various metabolic processes. Thus, to investigate the impact of NRF3 on metabolic control, we performed a metabolome analysis of HCT116-siNRF3 and siCtrl cells or H1299-oeNRF3/oeGFP cells. First, HCT116-siNRF3 and siCtrl cells were cultured with amino acid stimulation, whereas H1299-oeNRF3 and oeGFP cells were cultured without amino acid stimulation. Then, we obtained metabolite data of each cell in triplicate (Tables S1 and S2). K-means clustering and principal component analysis (PCA) for the quality control validation showed good reproduction between triplicate samples of each cell (Figures 5A and S5A). Subsequently, we performed a significance metabolomics analysis to identify metabolites with statistically significant changes in intracellular levels (Figure S5B). Results showed an alteration of 82 metabolites in HCT116-siNRF3 cells, compared with HCT116-siCtrl cells, and the alteration of 120 metabolites in H1299-oeNRF3 cells, compared with H1299-oeGFP cells (Tables S3 and S4). Among these metabolites, 55 were common (Figure 5B) and strongly enriched in several glucose metabolic pathways related to the Warburg effect, including glycolysis, the pentose-phosphate pathway (PPP), and the tricarboxylic acid cycle (TCA) (Figures 5C and 5D), suggesting the potential role of NRF3 in glucose metabolism reprogramming.

**Figure 6:**
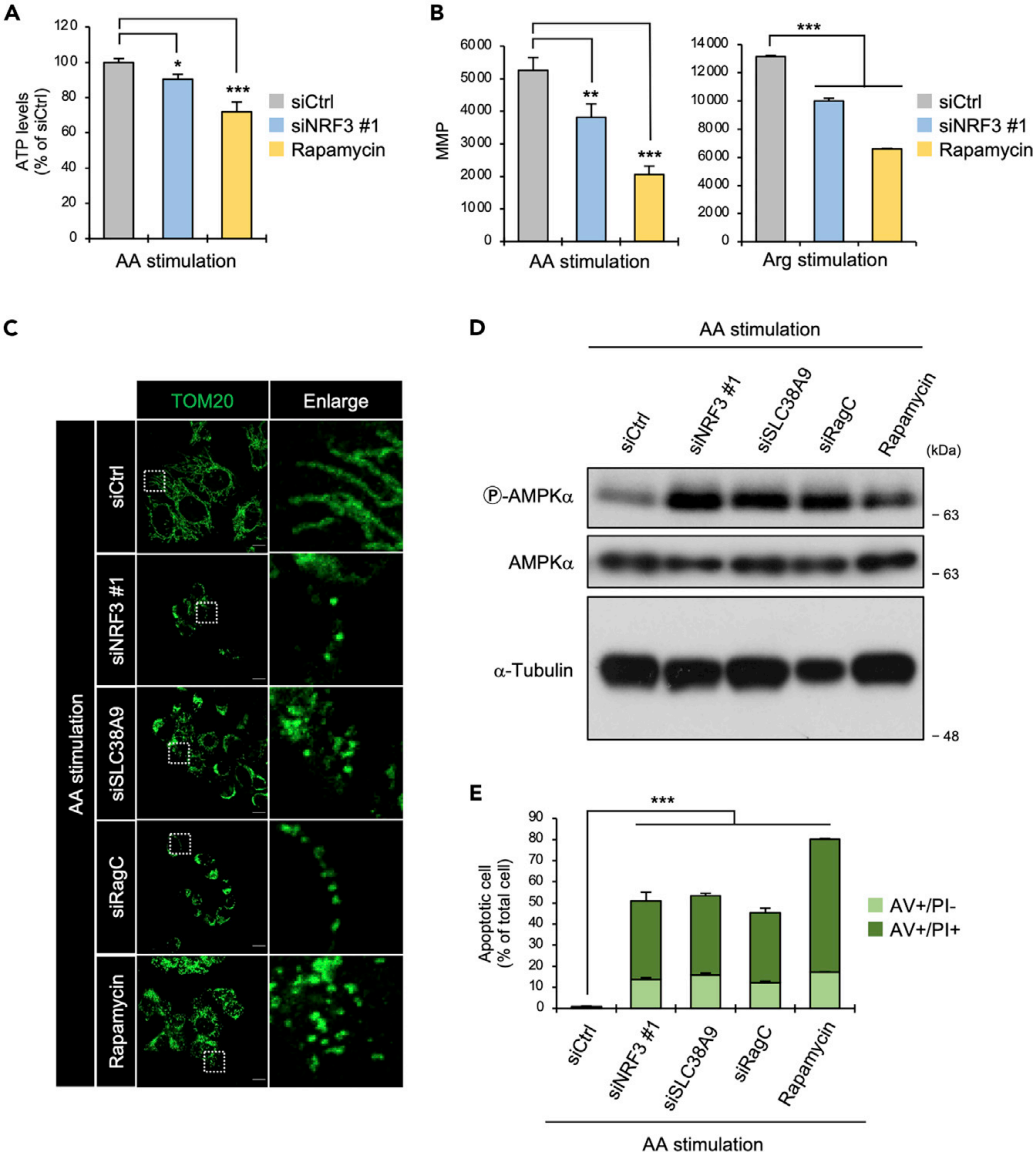
NRF3–mTORC1 axis inhibition causes mitochondrial defect and apoptosis induction (A) The effect of NRF3 knockdown or mTORC1 inhibition on intracellular ATP content in response to amino acid stimulation. HCT116 cells were transfected with the indicated siRNA for two days. Then, the cells were cultured without all amino acids for 5 h, after which they were restimulated with all amino acids for 15 min. Next, as a control, HCT116-siCtrl cells were treated with amino acid stimulation in addition to 10 μM rapamycin. (B) The effect of NRF3 knockdown or mTORC1 inhibition on mitochondrial membrane potential in response to amino acid stimulation (left) or arginine stimulation (right). HCT116 cells were transfected and stimulated as in (A). Then, the cells were stained with CMXRos, after which median fluorescence intensity (MFI) values were measured by flow cytometry. (C) The effect of indicated gene knockdown or mTORC1 inhibition on mitochondrial morphology in response to amino acid stimulation. HCT116 cells were transfected and stimulated as in (A). (D) The effect of indicated gene knockdown or mTORC1 inhibition on p-AMPKα levels in response to amino acid stimulation. HCT116 cells were transfected and stimulated as in (A). (E) The effect of indicated gene knockdown or mTORC1 inhibition on apoptosis in response to amino acid stimulation. HCT116 cells were transfected and stimulated as in (A). Then, the cells were stained with annexin V (AV) and PI for visualization. (A, B, E) ANOVA followed by Tukey’s test: ∗∗∗p< 0.005; ∗∗p< 0.01; ∗p< 0.05 (n = 3, Mean ± SD). See also Figure S6.

The Warburg effect affects mitochondrial ATP production in cancer cells.^40^ Therefore, to validate the potential role of NRF3 in ATP production under amino acid stimulation, we measured the ATP content in cells using a luciferase luminescence-based assay. Under amino acid stimulation, intracellular ATP levels were reduced by NRF3 knockdown, similar to treatment with the mTORC1 inhibitor, rapamycin (Figure 6A). Subsequently, we investigated the effect of NRF3 knockdown on mitochondrial membrane potential (MMP), which is critical for ATP production. Notably, CMXRos is a lipophilic cationic fluorescent dye that is concentrated inside the mitochondria based on their negative MMPs. With this MMP indicator, we showed that NRF3 knockdown or rapamycin treatment reduced the fluorescence intensity of CMXRos under amino acid or arginine stimulation (Figure 6B). Also, mitochondrial morphology was impaired by NRF3 knockdown or rapamycin treatment (Figures 6C and S6A, siCtrl versus siNRF3, siCtrl versus rapamycin). Of interest, similar results were observed with *SLC38A9* and *RagC* knockdown (Figure 6C, siCtrl versus siSLC38A9, siCtrl versus siRagC). Consistent results were further obtained in which the knockdown of these genes or rapamycin treatment increased the phosphorylation levels of AMPK (p-AMPK) (Figure 6D). Because mitochondrial defects cause apoptosis,^41^ we then detected the apoptotic cells by flow cytometry using annexin V-FITC (AV) and propidium iodide (PI). As expected, NRF3 knockdown increased early apoptotic (AV+/PI-) and late apoptotic/necrotic cells (AV+/PI+) under amino acid stimulation. Similar results were obtained by *SLC38A9* or *RagC* knockdown and rapamycin treatment (Figure 6E). Hence, these results suggest that NRF3 maintains mitochondrial function to adapt the cells to fluctuating amino acids in the extracellular environment.

Furthermore, the knockdown of *NRF3*, *SLC38A9*, or *RagC* and the treatment with rapamycin defected mitochondrial morphology and increased p-AMPK levels without any amino acid stimulation (Figures S6B and S6C). With the above insight that NRF3 is implicated in the basal activity of mTORC1 by expressing these genes under normal conditions (Figures 1E and S3B–S3H), our results imply that mitochondrial regulation through the NRF3–mTORC1 axis occurred in cancer cells under normal conditions. Mitochondria are highly dynamic organelles undergoing coordinated cycles of fission and fusion, referred to as mitochondrial dynamics.^42^ Mitochondrial dynamics are regulated by several key factors, such as Mitofusin 1 (MFN1), Mitofusin 2 (MFN2), Optic Atrophy 1 (OPA1), and Dynamin-related protein 1 (DRP1).^42^ However, NRF3 knockdown did not affect the expression of mitochondria-shaping protein genes (Figure S6D). Similarly, NRF3 knockdown did not change the protein levels of Mitochondrial Fission Process 1 (MTFP1), which is increased by mTORC1 activation.^43^ Meanwhile, we observed that NRF3 knockdown and rapamycin treatment increased the phosphorylation levels of DRP1 at S637 (p-DRP1 (S637)) (Figure S6E), attenuating the DRP1-mediated process of mitochondrial fission.^44^^,^^45^ These results suggest that DRP1 phosphorylation is implicated in mitochondrial regulation by the NRF3–mTORC1 axis.

### Aberrant upregulation of the NRF3–mTORC1 axis causes tumor growth and is associated with poor prognosis

**Figure 7:**
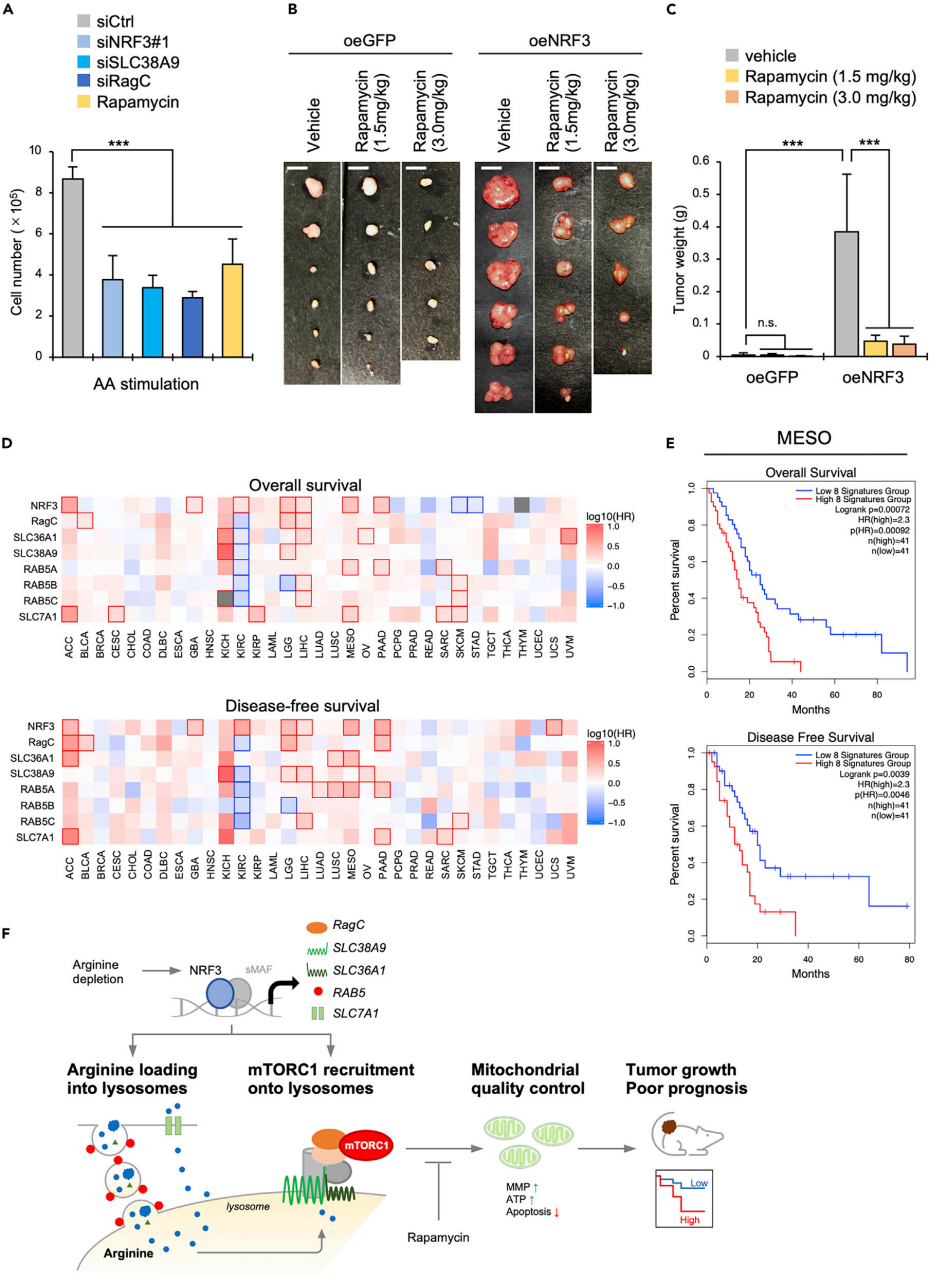
NRF3-mTORC1 axis aberration causes tumor growth and poor prognosis (A) The effect of indicated gene knockdown or mTORC1 inhibition on the *in vitro* growth of cancer cells under amino acid stimulation. HCT116 cells were transfected with the indicated siRNA for two days. Next, the cells were cultured without all amino acids for 5 h, then restimulated with all amino acids for 15 min. Subsequently, the cell numbers were counted using a hemocytometer. As a control, HCT116-siCtrl cells were treated with 10 μM rapamycin for two days and amino acid stimulation (n = 3, Mean ± SD). (B and C) The effect of mTORC1 inhibition on NRF3-dependent tumor growth in mouse xenograft models. H1299-oeGFP and H1299-oeNRF3 cells were injected subcutaneously into BALB/cAJcl-Foxn1^nu^ mice, and then, rapamycin (1.5 mg/kg or 3.0 mg/kg) was intraperitoneally administered once every two days. Photographs and weights of tumors four weeks after injection are shown in (B) and (C). In (B), Scale bars, 5 mm. (n = 5–6, Mean ± SD). (D) Overall or disease-free survival significance maps of the eight indicated genes in various cancer types, estimated using the Mantel–Cox test with a Hazard ratio (HR). (E) Kaplan–Meier plots comparing overall or disease-free survival of mesothelioma (MESO) patients with lower or higher expression levels of eight signatures, involving *NRF3, RagC, SLC36A1, SLC38A9, RAB5A RAB5B, RAB5C,* and *SLC7A1* genes. (F) A schematic showing NRF3-mediated arginine-dependent mTORC1 activation for cancer development. See the discussion section for details. (A, C) ANOVA followed by Tukey’s test: ∗∗∗p< 0.005; n.s., not significant. See also Figure S7.

Consistent with the apoptosis assay results (Figure 6E), we observed that the *in vitro* growth of cancer cells under amino acid stimulation was reduced by the knockdown of *NRF3*, *SLC38A9,* or *RagC* and by rapamycin treatments (Figure 7A). Similar results were obtained under arginine stimulation (Figure S7A). Therefore, to confirm the impact of the NRF3–mTORC1 axis on *in vivo* tumorigenesis, we performed xenograft experiments using H1299-oeNRF3 and oeGFP cells. As a result, the NRF3-dependent tumor growth was suppressed by rapamycin administration (Figures 7B, 7C, andS7B). Finally, we validated the clinical relevance between the prognosis of patients with cancer and the expression of genes focused on in this study. Among the 33 types of cancer, the overall survival or disease-free survival rates for patients with adrenocortical carcinoma (ACC), brain lower-grade glioma (LGG), liver hepatocellular carcinoma (LIHC), mesothelioma (MESO), or pancreatic adenocarcinoma (PAAD) were negatively correlated with higher expression levels of the *NRF3* gene, including two or more genes among *SLC36A1, SLC38A9*, *RagC*, each *RAB5* isoforms, and *SLC7A1* genes (Figure 7D). Similarly, the Kaplan–Meier plots of these cancer patients showed a poor prognosis with higher expression levels of *NRF3*, *SLC36A1, SLC38A9*, *RagC*, each *RAB5* isoforms, and *SLC7A1* genes (Figures 7E and S7C). These results provide the pathophysiological potential of the NRF3–mTORC1 axis for cancer development.

## Discussion

Cancer cells trigger mTORC1 activation, which promotes adaptation to extracellular environmental changes and rapid growth. Here, we showed the arginine-dependent activation mechanism of mTORC1 by a CNC-family transcription factor, NRF3, for cancer development (Figure 7F). Once arginine is depleted, NRF3 induces directly and indirectly the gene expression of *RagC, SLC38A9,* and *SLC36A1*, resulting in the recruitment of mTORC1 onto lysosomes in response to arginine restimulation. Then, to arginine-dependently activate mTORC1, NRF3 also enhances arginine loading into lysosomes through RAB5-mediated macropinocytosis and SLC7A1-mediated transport. Furthermore, metabolome analysis suggests a potential role of NRF3 in glucose metabolism reprogramming, related to the Warburg effect. Besides, inhibiting the NRF3–mTORC1 axis causes intracellular ATP reduction and apoptosis induction through mitochondrial membrane potential defect. Consistently, the aberrant upregulation of the NRF3–mTORC1 axis promotes tumor growth and predicts poor prognosis. In conclusion, this study sheds light on the physiological aspects of NRF3 in arginine-dependent mTORC1 activation for tumorigenesis. Also, we discussed the impacts of these findings on the arginine addiction of cancer cells and mitochondrial quality control. Then, we described the possible activation mechanism of NRF3 by arginine and the complicated regulation of mTORC1 signals by NRF1, NRF2, and NRF3.

Arginine is a nonessential amino acid required to synthesize creatine, polyamines, nitric oxide, and urea.^46^ Many human cells acquire sufficient levels of arginine from food, which are also synthesized from aspartate and citrulline through the urea cycle.^47^ However, accumulating evidence suggests that some cancer cells cannot synthesize sufficient arginine.^48^ For example, the *in vitro* and *in vivo* growth of hepatocellular carcinoma and melanoma are reduced by treating arginine deiminase (ADI), a metabolic enzyme that converts arginine to citrulline.^49^ As a result, these recombinant ADI proteins are currently in clinical trials to treat arginine auxotrophic hepatocellular carcinomas, melanomas, and several tumors.^50^ These insights indicate the high dependency of arginine on cancer cell proliferation. Also, this study discovered that NRF3 enhances arginine loading into lysosomes for mTORC1 activation and mitochondrial quality control through both macropinocytosis and transport (Figures 4, 5, and 6). This enhancement results in the aberrant upregulation of the NRF3–mTORC1 axis to promote tumor growth and predict poor prognosis (Figure 7). Thus, our findings suggest that cancer cells can prompt NRF3 to enhance arginine uptake for mTORC1 activation and mitochondrial quality control. Further studies are needed to clarify this issue.

Mitochondria are not only the leading powerhouse of the cell, generating ATP, but also the hubs of cell survival and apoptosis.^41^ Mitochondrial quality control systems include fusion and fission, coordinated by cell growth and division.^42^ In this study, we observed that *NRF3* knockdown and rapamycin treatment impaired mitochondrial function and caused mitochondrial fragmentation under amino acid stimulation (Figures 6A–6C), agreeing with a previous insight into the direct control of mitochondrial function by mTOR.^51^ Also, NRF3 knockdown and rapamycin treatment increased apoptotic cells (Figure 6E) and phosphorylated DRP1 proteins at Ser637 (Figure S6E), disturbing mitochondrial fission progression.^44^^,^^45^ Although MTFP1 is a critical regulator of DRP1 phosphorylation downstream of the mTORC1 signaling pathway,^43^ our investigations showed that NRF3 knockdown and mTORC1 inhibition did not alter MTFP1 protein levels (Figure S6E), implying that NRF3-mediated mTORC1 activation inhibits mitochondrial fusion in a DRP1 phosphorylation-dependent and MTFP1 expression-independent manner. Mitophagy is another mitochondrial quality control system that responds to mitochondrial fusion loss and mitochondrial fission induction.^52^ Recently, our other studies showed that NRF3 also activates autophagy (manuscript under submission). These insights suggest that NRF3-mediated mTORC1 activation uses mitophagy for mitochondrial quality control. Moreover, other groups reported that DRP1 interacts with BAX and induces apoptosis,^53^ implying the unknown effect of siNRF3-increased DRP1 phosphorylation on the interaction with apoptotic proteins. A future study should address these points.

In addition, this study suggests that the depletion of amino acids activates NRF3, particularly arginine (Figures 3A and S2C). Amino acid depletion promotes the homodimerization of DDI2ΔUBL (Figures S2D and S2E). Specifically, DDI2ΔUBL contains the RVP domain, whose homodimer forms the catalytic site.^34^ Overall, arginine is implicated in the RVP homodimerization for NRF3 activation, although its structural basis remains unclear. Therefore, to discuss this issue, we focused on previous HIV-1 protease (PR) studies structurally similar to DDI2-RVP. One study used was electrospray ionization mass spectrometry (ESI-MS) analysis using a recombinant protein.^54^ The termini interface comprised antiparallel β-sheets involving the catalytic site’s N- and C-termini residues. ESI-MS results showed the two-step processes of PR dimerization: initial weak interactions between the catalytic site interface and subsequent interactions between the termini interface. Furthermore, other studies indicated the significance of cysteine residues in PR conserved in DDI2-RVP for PR activity. Of interest, the PR activity was regulated through cysteine modification by glutathione or oxidation effects.^55^^,^^56^ Hence, since arginine supplementation induces glutathione synthesis,^57^ these insights suggest that arginine depletion enhances DDI2 homodimerization through cysteine modification by glutathione, resulting in protein processing and the transcriptional activation of NRF3.

Unlike NRF3, NRF1 and NRF2 are activated by mTORC1. mTORC1 stimulates a transcriptional program involving the sterol-regulatory element-binding protein 1 (SREBP1), subsequently inducing the gene expression of NRF1 and promoting the production of more proteasomes.^11^ Also, mTORC1 leads to the phosphorylation of the autophagy-adaptor protein, p62, inducing the autophagic degradation of kelch-like ECH-associated protein 1 (KEAP1), an adaptor of the CUL3-ubiquitin E3 ligase complex, responsible for degrading NRF2. These investigations result in NRF2 activation and hepatocellular carcinoma cell growth.^58^ Previously, we reported that NRF3 represses NRF1 translation by inducing the gene encoding the translational regulator, cytoplasmic polyadenylation element-binding protein 3 (CPEB3).^59^ In addition, NRF3 also reprograms lipid metabolism through the transcriptional activation of SREBP2,^10^ which is coupled with SREBP1.^60^ Similarly, this study revealed NRF3-mediated mTORC1 activation in response to amino acid stimulation. These insights imply the complicated network regulation of the mTORC1 signal by these three NRFs. Moreover, two-independent transcriptome studies suggested that NRF3 controlled a considerably smaller transcriptional network, compared with NRF1 and NRF2.^61^^,^^62^ Of interest, NRF3 is not broadly expressed across normal tissues like NRF1 and NRF2. Meanwhile, NRF3 displays uneven tumor tissue distribution, implying that NRF3 activates a cancer-specific transcriptional program in response to tumor microenvironmental factors, such as amino acids.

### Limitations of the study

This study was restricted to *in vitro* cell experiments. Consequently, it is essential to determine whether our findings contribute to the *in vivo* phenotype, specifically the effect of arginine stimulation on NRF3-mediated mTORC1 activation and cancer development. Future research must also elucidate the molecular mechanism underlying the NRF3 activation through DDI2 heterodimerization in response to arginine stimulation and the mitochondrial quality control by NRF3 activation through DRP1 phosphorylation. Our previous study identified RAB5A, B, and C as NRF3-target genes.^10^ Meanwhile, this study showed that the gene expression of RAB5B and C, except RAB5A, were significantly reduced by NRF3 knockdown under arginine stimulation (Figure S4D). These results imply the distinct target of NRF3 between normal and arginine-stimulation conditions, although the future study is needed.

## STAR★Methods

### Key resources table

**Table:** 

REAGENT or RESOURCE	SOURCE	IDENTIFIER
**Antibodies**		

Rabbit monoclonal phospho-p70 S6 kinase (Thr389) (108D2) antibody	Cell Signaling Technology	Cat# 9234; RRID:AB_2269803
Rabbit monoclonal p70 S6 kinase (49D7) antibody	Cell Signaling Technology	Cat# 2708; RRID:AB_390722
Rabbit polyclonal phospho-Akt (S473) antibody	Cell Signaling Technology	Cat# 9271; RRID:AB_329825
Rabbit monoclonal Akt (pan) (C67E7) antibody	Cell Signaling Technology	Cat# 4691; RRID:AB_915783
Rabbit monoclonal mTOR (7C10) antibody	Cell Signaling Technology	Cat# 2983; RRID:2105622
Mouse monoclonal LAMP-1 (H4A3) antibody	Santa Cruz Biotechnology	Cat# sc-20011; RRID:AB_626853
Rabbit polyclonal Sestrin2 antibody	Proteintech	Cat# 10795-1-AP; RRID:AB_218540
Mouse monoclonal FLAG M2 antibody	Sigma-Aldrich	Cat# F1804; RRID:AB_262044
Rabbit polyclonal SLC38A9 antibody	Sigma-Aldrich	Cat# HPA043785; RRID:AB_10961859
Rabbit monoclonal RagC (D8H5) antibody	Cell Signaling Technology	Cat# 9480; RRID:AB_10614716
Rabbit polyclonal SLC36A1 antibody	Abcam	Cat# ab189441
Mouse monoclonal TOM20 (F-10) antibody	Santa Cruz Biotechnology	Cat# sc-17764; RRID:AB_628381
Rabbit monoclonal phospho-AMPKα (Thr172) (40H9) antibody	Cell Signaling Technology	Cat# 2535; RRID:AB_331250
Rabbit polyclonal AMPKα antibody	Cell Signaling Technology	Cat# 2532; RRID:AB_330331
Rabbit monoclonal phospho-DRP1 (S637) (D3A4) antibody	Cell Signaling Technology	Cat# 6319; RRID:AB_10971640
Rabbit monoclonal DRP1 (D6C7) antibody	Cell Signaling Technology	Cat# 8570; RRID:AB_10950498
Rabbit polyclonal MTP18 antibody	Abcam	Cat# ab198217
Mouse human NRF3 (#9408) antibody	RIKEN-BRC	RCB4901
Mouse monoclonal α-Tubulin antibody	Sigma-Aldrich	Cat# T9026; RRID:AB_477593
Mouse monoclonal GFP (B-2) antibody	Santa Cruz Biotechnology	Cat# sc-9996; RRID:AB_627695
Goat anti-Rabbit IgG (H+L) Secondary antibody, HRP	Thermo Fisher Scientific	Cat# 65-6120; RRID:AB_2533967
Goat anti-Mouse IgG (H+L), horseradish peroxidase conjugate	Thermo Fisher Scientific	Cat# G-21040; RRID:AB_2536527
Goat anti-Rabbit IgG (H+L), Alexa Fluor 488	Thermo Fisher Scientific	Cat# A-11034; RRID:AB_2576217
Goat anti-Mouse IgG (H+L), Alexa Fluor 546	Thermo Fisher Scientific	Cat# A-11030; RRID:AB_2534089
Goat anti-Mouse IgG (H+L), Alexa Fluor 488	Thermo Fisher Scientific	Cat# A-11029; RRID:AB_2534088

**Chemicals, peptides, and recombinant proteins**		

ANTI-FLAG M2 affinity gel	Sigma-Aldrich	A2220
Rapamycin	LC Laboratories	R-5000
EIPA	Cayman	14406
FITC-BSA	Thermo Fisher Scientific	A23015
BSA	Sigma-Aldrich	A1470
MitoTracker Red CMXRos	Thermo Fisher Scientific	M7512
PNGase F	New England Biolab	P0704
MG-132	Peptide Institute	3175-v
CultureSure L-Glutamine, Animal-derived-free	Wako Pure Chemical Industries	035-23611
CultureSure L-Leucine, Animal-derived-free	Wako Pure Chemical Industries	031-23711
CultureSure L-Arginine Hydrochloride, Animal-derived-free	Wako Pure Chemical Industries	038-23601

**Critical commercial assays**		

"Cell" ATP Assay reagent	TOYOBO	CA10
MEBCYTO Apoptosis Kit (Annexin V-FITC Kit)	MBL International	4700

**Experimental models: Cell lines**		

Human: HCT116	RIKEN-BRC	RCB-2979
Human: H1299	ATCC	N/A
Human: DLD-1	Cell Resource Center for Biomedical Research, Institute of Development, Aging and Cancer Tohoku University	TKG0379
Human: PK-45H	RIKEN-BRC	RCB1973

**Experimental models: Organisms/strains**		

Mouse: BALB/cAJcl-Foxn1nu	CLEA Japan	BALB/cAJcl-nu/nu

**Recombinant DNA**		

pMRX-IP-GFP-LC3-RFP-LC3ΔG	RIKEN-BRC	RDB14600
pRK5 FLAG-WDR24	Addgene	Cat# 46334; RRID:Addgene_46334
pRK5 FLAG-SLC38A9.1	Addgene	Cat# 71855; RRID:Addgene_71855
pRK5 FLAG-RagC	Addgene	Cat# 99723; RRID:Addgene_99723
pBiFC-VN173	Addgene	Cat# 22010; RRID:Addgene_22010
pBiFC-VC155	Addgene	Cat# 22011; RRID:Addgene_22011
pcDNA3.1	Thermo Fisher Scientific	N/A
p3×FLAG-CMV 10 harboring the full-length EGFP gene	Waku et al.^7^	https://doi.org/10.1128/MCB.00597-19
pcDNA3.1 harboring FLAG-tag sequence	N/A	N/A
pcDNA3.1 harboring HA-tag sequence	N/A	N/A

**Software and algorithms**		

GSEA v.4.1.0	Subramanian et al.^27^	https://www.gsea-msigdb.org/gsea/
UCSC Genome Browser	Kent et al.^63^	https://genome.ucsc.edu/
MetaboAnalyst	Xia et al.^64^	https://www.metaboanalyst.ca/
GEPIA2	Tang et al.^65^	http://gepia2.cancer-pku.cn/

**Deposited data**		

Raw and analyzed data	Waku et al.^10^	GEO: GSE176444

### Resource availability

#### Lead contact

Further information and requests for resources and reagents should be directed to and will be fulfilled by the lead contact, Tsuyoshi Waku (twaku@mail.doshisha.ac.jp).

#### Materials availability

All reagents will be made available on request after completion of a Material Transfer Agreement.

### Experimental model and subject details

#### Cell lines

HCT116 and DLD-1 cells were cultured in DMEM/high glucose medium (Wako Pure Chemical Industries). H1299 and PK-45H cells were cultured in an RPMI-1640 medium (Nacalai Tesque). All media were supplemented with 10% FBS (Nichirei Biosciences), 40 μg/mL streptomycin, and 40 units/mL penicillin (Wako Pure Chemical Industries). All cell lines were cultured at 37°C with 5% CO_2_ incubator. H1299 stably expressing NRF3 and GFP cells were generated previously.^7^ To generate HCT116 cells stably expressing the GFP-LC3-RFP-LC3ΔG probe, HCT116 cells were transfected with pMRX-IP-GFP-LC3-RFP-LC3ΔG^28^ and selected with 1 μg/mL puromycin. The plasmid was provided by the RIKEN BRC through the National BioResource Project of the MEXT/AMED, Japan.

#### Tumor xenograft mice model

This assay was based on a previous method.^7^ H1299-oeNRF3 or H1299-oeGFP cells were subcutaneously injected into both flanks of 4-week-old female BALB/cAJcl-Foxn1^nu^ mice (CREA Japan). After one week, rapamycin (LC Laboratory) was administered intraperitoneally once every two days at 1.5 or 3.0 mg/kg. Tumor growth was monitored by measuring the tumor size using calipers. Tumor volume was determined using the formula V = 1/2 × larger diameter × (smaller diameter)^2^. After three weeks, tumor tissues were removed, and the weights were measured. All animal experiments were performed in accordance with the guidelines for the care and use of laboratory animals at Doshisha University, Japan.

### Method details

#### Transfection

Transfection of plasmid DNA and siRNA was performed using polyethylenimine and RNAiMAX (Invitrogen), respectively. The sequences of the siRNA oligonucleotides are listed in Table S5.

#### Immunoblotting

To extract NRF3 protein, cells were lysed with 1×SDS sample buffer (50mM Tris-HCl [pH6.8], 10% glycerol, 1% SDS) and sonicated for breaking DNA and boiled with 2% 2-mercaptoethanol at 95°C for 5 min. To extract the other proteins, cells were lysed with RIPA buffer (20 mM Tris-HCl [pH 7.6], 50mM NaF, 10mM NaCl, 10mM sodium pyrophosphate, 10mM β-glycerophosphate, 1mM EDTA, 10% glycerol, 1% NP-40, protease inhibitor cocktail). The lysates were centrifuged at 4°C and 13,000 rpm for 10 min for debris removal and boiled with 5×SDS sample buffer and 2% 2-mercaptoethanol at 95°C for 5 min. For detection of SLC36A1 and SLC38A9 proteins, the lysates were incubated with PNGase F (New England Biolabs) before addition of 5×SDS sample buffer and skipped 95°C boiling step. The protein quantities were measured using a BCA kit (Wako Pure Chemical Industries). The proteins were separated with SDS-PAGE and transferred to PVDF membranes (Sigma-Aldrich). After blocking with appropriate solution at room temperature for 1 h, the membranes were incubated with primary antibody solution at 4°C overnight and washed with TBS-T (20 mM Tris–HCl [pH 7.6], 137 mM NaCl, and 0.1% Tween20). The membranes then were incubated with a horseradish peroxidase-conjugated secondary antibody (Invitrogen) and washed with TBS-T and developed with enhanced chemiluminescence (GE Healthcare). Representative blots from independent experiments were shown.

#### Immunostaining

Cells were fixed with 4% formaldehyde for 15 minat 37°C, washed three times with PBS, and permeabilized with 0.3% Triton X-100 goat serum in PBS for 1 hat room temperature. The cells were washed three times with PBS and treated with the antibodies indicated in the figures for 1 day 4°C. After the cells were washed three times with PBS, they were incubated with Alexa Fluor 488-, Alexa Fluor 546-, Alexa Fluor 594- or Alexa Fluor 647-conjugated secondary antibodies (Invitrogen) for 1 hat room temperature. The nuclei were stained with 4′,6′-diamidino-2-phenylindole (DAPI) (Dojindo Molecular Technologies). After washing with PBS three times, the cells were sealed with a fluorescence mounting medium (Dako). Fluorescent images were acquired with a confocal microscope (LSM900, Zeiss) and representative images were shown. Colocalization analysis was performed with an ImageJ plugin JACoP using Mander’s coefficient.^66^

#### RNA extraction and RT-qPCR

This assay was based on a previous method.^10^ The extraction and purification of total RNA were using ISOGEN II (NIPPON GENE) according to the manufacturer’s protocol. Aliquots of 1 μg total RNA were reverse transcribed using pd (N)6 random primer (Takara Bio) and Moloney murine leukemia virus reverse transcriptase (Invitrogen) with a 250 mM deoxy nucleoside triphosphate (Takara Bio), according to the manufacturer’s instructions. RT-qPCR was also performed using SYBR Premix Ex Taq II (Takara Bio), and primers for genes were conducted using a Thermal Cycler Dice Real-Time System (Takara Bio). Each gene expression level in human cells was also normalized to the mRNA levels of the human β-actin gene. qPCR primer sequences are described in Table S5.

#### ChIP-qPCR

This assay was based on a previous method.^10^ Cells were treated with 1 μM MG-132 (Peptide Institute) for 16 h. The cells were fixed with 1% formaldehyde for 10 min at room temperature, and then, glycine was added to make a final concentration of 0.125 M. The cells were then lysed using a cell lysis buffer (5 mM Tris–HCl [pH 8.0], 85 mM KCl, and 0.5% NP-40) with protease inhibitors (Nacalai Tesque) and then centrifuged at 2,000 rpm at 4°C for 3 min. The pellets were further lysed using a nuclei lysis buffer (50 mM Tris–HCl [pH 8.0], 10 mM EDTA, and 1% SDS) with protease inhibitors, after which the lysates were sonicated. After centrifugation at 15,000 rpm at 8°C for 10 min, the supernatants were collectedand then diluted in a ChIP dilution buffer (16.7 mM Tris–HCl [pH 8.0], 167 mM NaCl, 1.2 mM EDTA, 1.1% TritonX-100, and 0.01% SDS). Also, the diluted samples were precleared with 20 μl of Dynabeads Protein G (ThermoFisher Scientific); then, the supernatants (used as an input sample) were incubated with 3 μg of anti-NRF3 antibody (RCB4901).^5^ The immunocomplexes were also collected by incubation with 20 μL of Dynabeads Protein G (ThermoFisher Scientific) and then washed with the following buffers: the low salt wash buffer (20 mM Tris–HCl [pH 8.0], 150 mM NaCl, 2 mM EDTA, 1% Triton X-100, and 0.1% SDS), high salt wash buffer (20 mM Tris–HCl [pH 8.0], 500 mM NaCl, 2 mM EDTA, 1% Triton X-100, and 0.1% SDS), and LiCl wash buffer (10 mM Tris–HCl [pH 8.0], 250 mM LiCl, 1 mM EDTA, 1% sodium deoxycholate, and 1% NP-40). Finally, the beads were washed twice with 1 ml of TE buffer (10 mM Tris–HCl [pH 8.0], and 1 mM EDTA). The immunocomplexes were then eluted by adding 100 μl of elution buffer (50 mM NaHCO_3_ and 1% SDS). After reverse cross-linking by adding 200 mM NaCl, the remaining proteins were digested by adding proteinase K. For quantification of NRF3 binding to the target regions, RT-qPCR was then performed using the purified DNA with the primers described in Table S5.

The ChIP-region in the *SLC38A9*, *RagC*, *SLC36A1*, or *SLC7A1* promoter at the human genome (GRCh37/hg19) was shown with ChIP-seq signals of MAFK. Multiple sequences of a candidate ARE in the indicated species were prepared using a web-tool UCSC Genome Browser.^63^

#### Co-IP experiment

This experiment was based on the previously described method.^24^ Cells were transfected with indicated plasmids. After one day, the cells were treated with indicated amino acid stimulation, and then lysed with Triton lysis buffer (40 mM HEPES [pH 7.4], 10mM β-glycerol phosphate, 10 mM sodium pyrophosphate, 2.5 mM MgCl_2_, 1% Triton X-100, and protease inhibitor cocktail), and then centrifuged at 4°C and 13,000 rpm for 10 min for debris removal. 50/50 slurry of FLAG-M2 affinity gel (Sigma-Aldrich) was rinsed three times with Triton lysis buffer, which were added into the cell lysates and then rotated at 4°C for 2 h. The lysates-gel mixtures were washed one time with Triton lysis buffer and three times with triton lysis buffer supplemented with 500 mM NaCl. The immunoprecipitated samples were denatured with an equal volume of 2×SDS sample buffer and were subjected to immunoblotting.

#### Autophagy flux

This assay was performed using a slight modification of a previously described method.^28^ HCT116 cells stably expressing the GFP-LC3-RFP-LC3ΔG probe were transfected with the indicated siRNA. At two days after the siRNA transfection, the cells were subjected to a fluorescent microscope (LX71, Olympus) to acquire the fluorescent images or a flow cytometry (FACSAriaII, BD Biosciences) to measure the median fluorescent intensity (MFI) values of GFP and RFP.

#### Macropinocytosis assay

This assay was based on a previous method.^10^ Briefly, cells were treated with 100 μM EIPA (Cayman) or DMSO and then incubated with 1 mg/ml FITC-BSA (Invitrogen) for 1 h. The cells were also washed twice with a FACS buffer (0.1% [w/v] sodium azide and 2% FBS in cold PBS). Then, the cells were resuspended in 500 μl FACS buffer, and the median fluorescent intensity (MFI) values were measured using a flow cytometer (FACSAriaII, BD Biosciences).

#### Apoptosis assay

This assay was conducted using MEBCYTO Apoptosis Kit (Annexin V-FITC Kit) (MBL International) according to the manufacturer’s protocol. Briefly, cells were collected, and resuspended in Binding buffer with Annexin V-FITC and propidium iodide (PI). Following incubation for 15 min at room temperature, the cells were subjected to dead-cell analysis using a flow cytometer (FACSAriaII, BD Biosciences).

#### BiFC method

The BiFC assay was performed as described previously.^67^ To generate plasmids expressing split Venus fragments-tagged DDI2ΔUBL, the DDI2 cDNA was cloned into pBiFC-VN173 (Addgene_22010) or pBiFC-VC155 (Addgene_22011), respectively. Both BiFC plasmids were co-transfected with a pmCherry-N1 plasmid (Clontech), which was used as an internal control. At one day after transfection, the cells were cultured without all amino acids for 3 h. The fluorescent intensities of Venus and mCherry were measured using a flow cytometer (FACSAriaII, BD Biosciences). Median fluorescence intensity (MFI) values of Venus in mCherry-positive cells were measured by flow cytometry.

#### Metabolome analysis

##### Instrumentation

All capillary electrophoresis time-of-flight mass spectrometry (CE-TOFMS) experiments were performed using Agilent 7100 CE capillary electrophoresis (Agilent Technologies), the Agilent 6230 LC/MSD TOF system (Agilent Technologies), an Agilent1100 series binary HPLC pump, and the G1603A Agilent CE-MS adapter- and G1607A Agilent CE-ESI-MS sprayer kit. For anionic metabolite analysis, the original Agilent stainless ESI needle was replaced with the Agilent G7100-60041 platinum ESI needle.^68^ System control and data acquisition were performed by Agilent MassHunter Workstation, and data analysis was done by Keio MasterHands software.

##### Cationic metabolite analysis

Separations were carried out in a fused silica capillary (50 μm i.d. x 100 cm total length) filled with 1 M formic acid as the electrolyte.^69^^,^^70^ Approximately 5 nl of sample solution were injected at 50 mbar for 5 sec and 30kV of voltage was applied. The capillary temperature was maintained at 20°C and the sample tray was cooled below 5°C. Methanol-water (50% v/v) containing 0.01 μM Hexakis(2,2-difluoroethoxy)phosphazene was delivered as the sheath liquid at 10 μl/min. Electrospray ionization (ESI)-time-of-flight mass spectrometry (TOFMS) was conducted in the positive ion mode and the capillary voltage was set at 4,000V. A flow rate of heated dry nitrogen gas (heater temperature 300°C) was maintained at 7 psig. In TOFMS, the fragmentor-, skimmer-, and Oct RFV voltage was set at 75V, 50V, and 500V, respectively. Automatic recalibration of each acquired spectrum was performed using reference masses of reference standards. The ^13^C isotopic ion of a protonated methanol dimer ([2MeOH+H]^+^, m/z 66.0631) and Hexakis(2,2-difluoroethoxy)phosphazene ([M+H]^+^, m/z 622.0290) provided the lock mass for exact mass measurements.^71^

##### Anionic metabolite analysis

A commercially available COSMO(+) (chemically coated with cationic polymer) capillary (50 μm i.d. x 105 cm total length) (Nacalai Tesque, Kyoto, Japan) was used with a 50 mM ammonium acetate solution (pH 8.5) as the electrolyte.^68^^,^^71^ Sample solution (30 nl) was injected at 50 mbar for 30 sec and −30kV of voltage was applied. Ammonium acetate (5 mM) in 50% methanol-water (v/v) containing 0.01 μM Hexakis(2,2-difluoroethoxy)phosphazene was delivered as the sheath liquid at 10 μl/min. ESI-TOFMS was conducted in the negative ion mode; the capillary voltage was set at 3,500V. For TOFMS, the fragmentor-, skimmer-, and Oct RFV voltage was set at 100V, 50V, and 500V, respectively. Automatic recalibration of each acquired spectrum was performed using reference masses of reference standards, i.e., ^13^C isotopic ion of deprotonated acetic acid dimer ([2CH_3_COOH-H]^-^, m/z 120.0384), and Hexakis + deprotonated acetic acid (m/z 680.03554) provided the lock mass for exact mass measurements.

#### Survival maps and for Kaplan–Meier analysis

These clinical analyses were performed using the GEPIA2, a web server for cancer and normal gene expression profiling and interactive analyses.^65^

### Quantification and statistical analysis

Data are reported as mean ± standard deviation (SD). Welch’s *t*-test and one-way analysis of variance (ANOVA) followed by Tukey’s post hoc test were used to compare two and multiple groups, respectively (∗∗∗p< 0.005; ∗∗p< 0.001; ∗p< 0.05; n.s., not significant). Statistical and enrichment analysis of metabolomics data are performed by MetaboAnalyst, a comprehensive platform dedicated for metabolomics data analysis via user-friendly, web-based interface.^64^

## Data Availability

The DNA microarray data have been deposited at NCBI’s Gene Expression Omnibus and are publicly available as of the date of publication. Accession numbers are listed in the key resources table. This paper does not report original code. Any additional information required to reanalyze the data reported in this paper is available from the lead contact upon request.

## Supplemental information

Document S1. Figures S1–S7

Table S1. Metabolomics data of HCT116-siCtrl and siNRF3 cells, related to Figure 5

Table S2. Metabolomics data of H1299-oeGFP and oeNRF3 cells, related to Figure 5

Table S3. Significance Analysis of Metabolomics between HCT116-siCtrl and siNRF3 cells, related to Figure 5

Table S4. Significance Analysis of Metabolomics between H1299-oeGFP and oeNRF3 cells, related to Figure 5

Table S5. qPCR primer and siRNA oligonucleotide sequences used in this study, related to STAR Methods
