# Supplementary material for: NRF3 activates mTORC1 arginine-dependently for cancer cell viability
Source: iScience. 2023 Jan 25;26(2):106045. doi: 10.1016/j.isci.2023.106045 (PMC9932127; doi:10.1016/j.isci.2023.106045)
Supplement: Document S1. Figures S1–S7 [file mmc1.pdf]

iScience, Volume *26*

Supplemental information

NRF3 activates mTORC1 arginine-dependently for cancer cell viability

Shuuhei Hirose, Tsuyoshi Waku, Misato Tani, Haruka Masuda, Keiko Endo, Sanae Ashitani, Iori Aketa, Hina Kitano, Sota Nakada, Ayaka Wada, Atsushi Hatanaka, Tsuyoshi Osawa, Tomoyoshi Soga, and Akira Kobayashi

Hirose et al. **Figure S1**

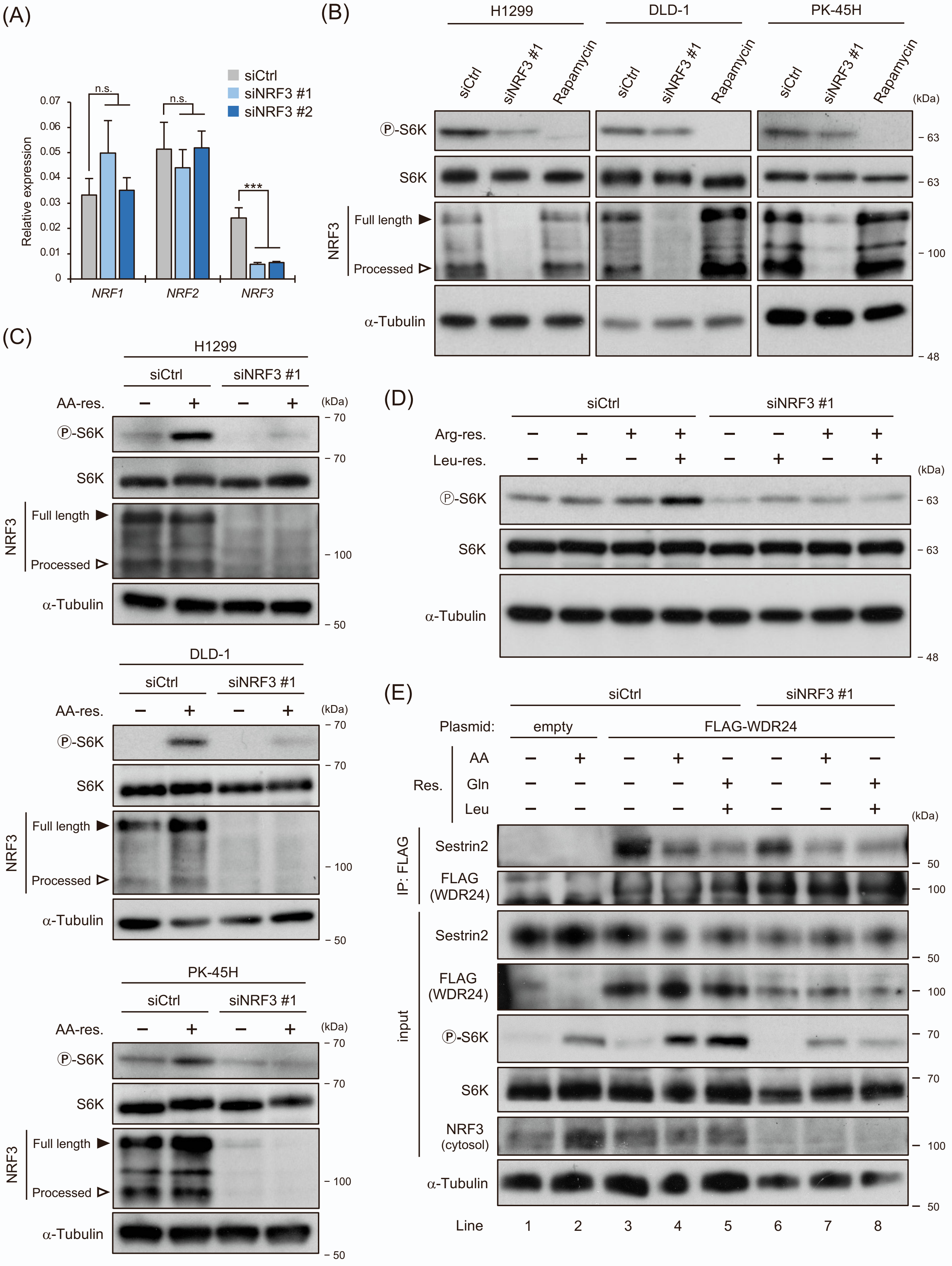

Figure S1. NRF3-mediated mTORC1 activation in various cancer cells, related to Figures 1 and 2

A. Knockdown specificity of siNRF3 for *NRF1* and *NRF2* mRNA. HCT116 cells were transfected with indicated siRNA for one day. The control siRNA is represented as siCtrl. ANOVA followed by Tukey's test: ***p < 0.005; n.s., not significant (n = 3, Mean ± SD). ***B.*** The effect of NRF3 knockdown on the basal activity of mTORC1 in various cancer cells. The indicated cells were transfected with indicated siRNA for one day. The control siRNA is represented as siCtrl. As a control, the cells were transfected with siCtrl and cultured with 10 µM rapamycin for one day. Full-length and processed NRF3 proteins are indicated with black and white arrowheads, respectively. ***C.*** The effect of NRF3 knockdown on mTORC1 activation in response to amino acid stimulation in various cancer cells. Indicated cells were transfected with the indicated siRNA for two days. Subsequently, H1299 and PK-45H cells were cultured without all amino acids for 16 h, then restimulated with or without all amino acids for 15 min (+/−AA-res.). Alternatively, DLD-1 cells were cultured without all amino acids for 16 h and then restimulated with or without all amino acids for one hour (+/−AA-res.). ***D.*** The effect of NRF3 knockdown on mTORC1 activation in response to arginine and/or leucine restimulation. HCT116 cells were transfected with the indicated siRNA for one day. Then, the cells were cultured without arginine and leucin for five hours and then restimulated with or without arginine (+Arg-res.) and leucin (+Leu-res.) for 15 min. ***E.*** The impact of NRF3 knockdown on Sestrin2-GATOR2 interaction in combination with leucine and arginine. HCT116 cells were transfected with the indicated siRNA for two days and then further transfected with pcDNA3.1 (empty) or pRK5 FLAG-WDR24 (FLAG-WDR24) plasmids. The cells were stimulated in as (D).

Hirose et al. Figure S2

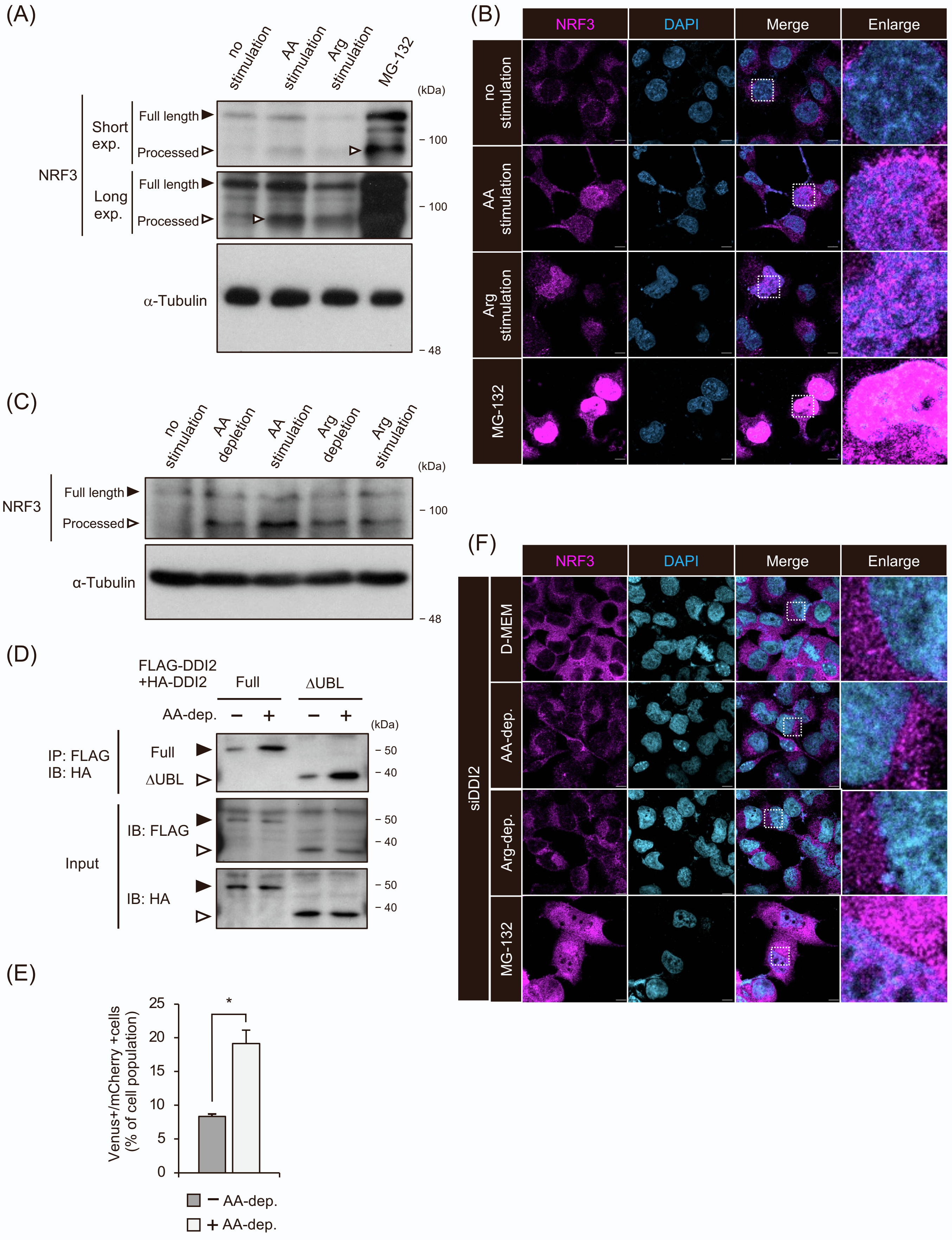

Figure S2. NRF3 processing and DDI2 dimerization with amino acid or arginine stimulation, related to Figure 3

A. The effect of amino acid or arginine stimulation on the protein processing of NRF3. For amino acid (AA) or arginine (Arg) stimulation, H1299-oeNRF3 cells were cultured without all amino acids or arginine for 24 h and then restimulated with all amino acids or arginine for 15 min (AA or Arg stimulation). As a positive control, the cells were incubated with 1 μM MG-132 for 24 h. ***B.*** The effect of amino acid or arginine stimulation on the nuclear localization of NRF3. For amino acid (AA) or arginine (Arg) stimulation, H1299-oeNRF3 cells were stimulated as in (A). Scale bars, 10 μm. ***C.*** The effect of amino acid or arginine depletion and stimulation on NRF3 protein processing. For amino acid (AA) or arginine (Arg) depletion, H1299-oeNRF3 cells were cultured without all amino acid or arginine for 24 h. For amino acid or arginine stimulation, the cells were stimulated as in (A). ***D.*** The effect of amino acid stimulation on the dimerization of DDI2 proteins. HCT116 cells were transfected with the expression plasmid harboring FLAG or HA-tagged full-length (Full, black arrowhead) or UBL-deleted DDI2 (ΔUBL, white arrowhead). Then, the cells were cultured with or without amino acid depletion (+/−AA-dep.) for three hours. ***E.*** The effect of amino acid stimulation on the dimerization of DDI2 proteins in living cells. HCT116 cells were transfected with the expression plasmid harboring split Venus N-terminal or C-terminal UBL-deleted DDI2 (ΔUBL) for one day and then stimulated as in (D). Finally, flow cytometry measured the median fluorescence intensity (MFI) values of Venus in mCherry-positive cells. Welch t-test: $*p < 0.05$ ($n = 3$, Mean ± SD). ***F.*** The effect of DDI2 knockdown on the nuclear localization of NRF3 in response to amino acid or arginine depletion. H1299-oeNRF3 cells were transfected with siDDI2 and stimulated as in Figure 3A. Scale bars, 10 μm.

Hirose et al. **Figure S3**

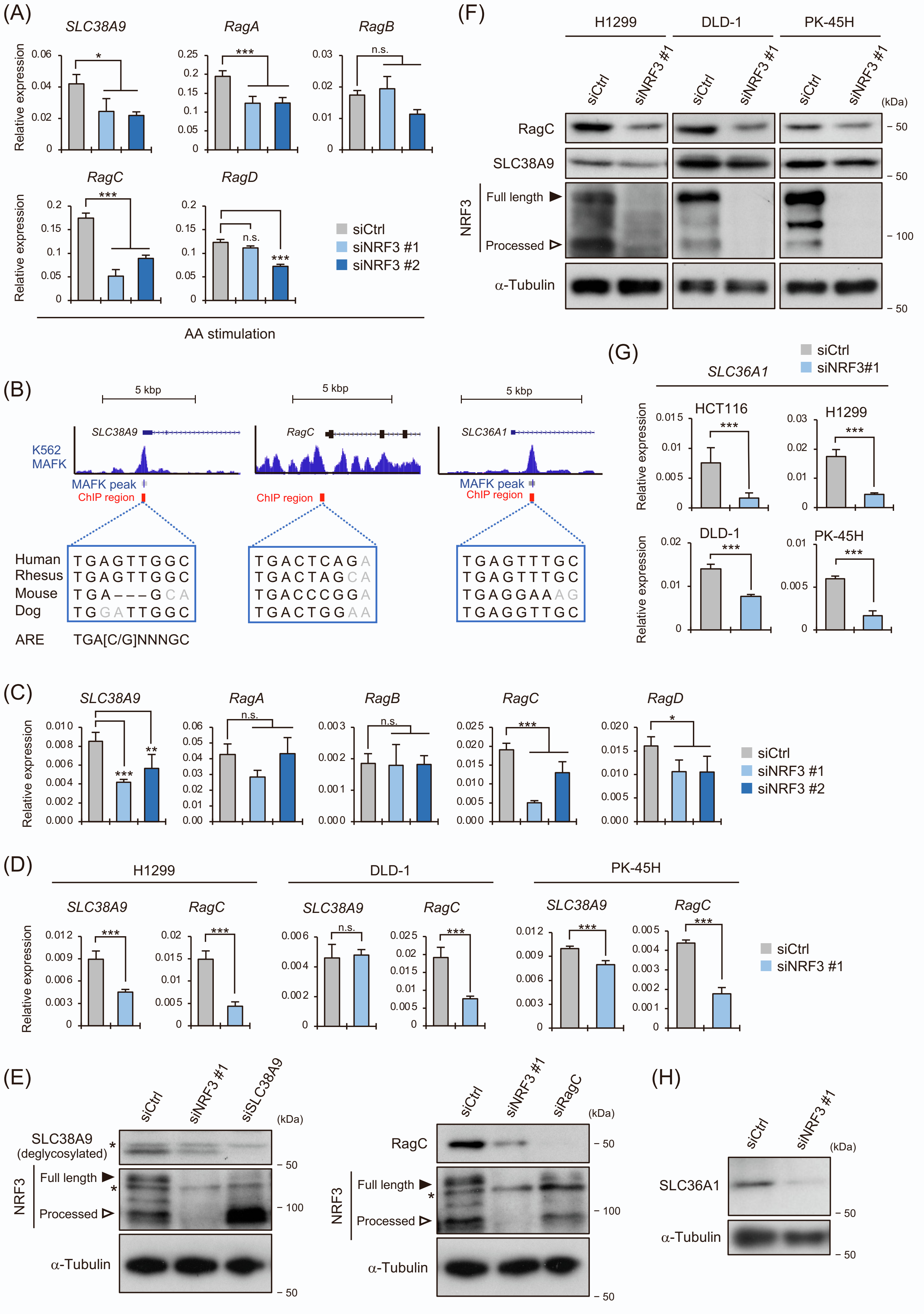

Figure S3. NRF3-mediated induction of *SLC38A9, RagC,* and *SLC36A1* expression without amino acid stimulation, related to Figure 3

A. The effect of NRF3 knockdown on the expression of five Ragulator-associated genes in response to amino acid stimulation. For two days, HCT116 cells were transfected with the indicated siRNA. The cells were cultured without all amino acids for five hours and then restimulated with all amino acids for 15 min. ***B.*** The genome locus of the *SLC38A9, RagC,* or *SLC36A1* promoter in the human genome (GRCh37/hg19) was shown using ChIP-seq signals of MAFK (blue histogram). Multiple sequences of a candidate ARE in the indicated species were prepared using a web-tool UCSC Genome Browser [60]. The region of ChIP-qPCR (ChIP region) is shown as a red rectangle. ***C.*** The effect of NRF3 knockdown on the basal mRNA levels of Ragulator-associated genes. HCT116 cells were transfected with the indicated siRNA for two days. ***D.*** The effect of NRF3 knockdown on the basal mRNA levels of *SLC38A9* and *RagC* genes in various cancer cells. The indicated cells were transfected with indicated siRNA for two days. ***E.*** The effect of the indicated gene knockdown on the basal protein levels of SLC38A9 and RagC. HCT116 cells were transfected with the indicated siRNA for two days. Non-specific bands are shown as (*). ***F.*** The effect of NRF3 knockdown on the basal protein levels of SLC38A9 and RagC in various cancer cells. The indicated cells were transfected with indicated siRNA for two days. ***G.*** The effect of NRF3 knockdown on the basal mRNA levels of *SLC36A1* in various cancer cells. The indicated cells were transfected with indicated siRNA for two days. ***H.*** The Effect of NRF3 knockdown on the basal protein levels of SLC36A1. HCT116 cells were transfected with the indicated siRNA for two days. (*D, G*) Welch *t*-test, (*A, C*) ANOVA followed by Tukey's test: ***$p < 0.005$; **$p < 0.01$; *$p < 0.05$; n.s., not significant ($n = 3$, Mean ± SD).

Hirose et al. **Figure S4**

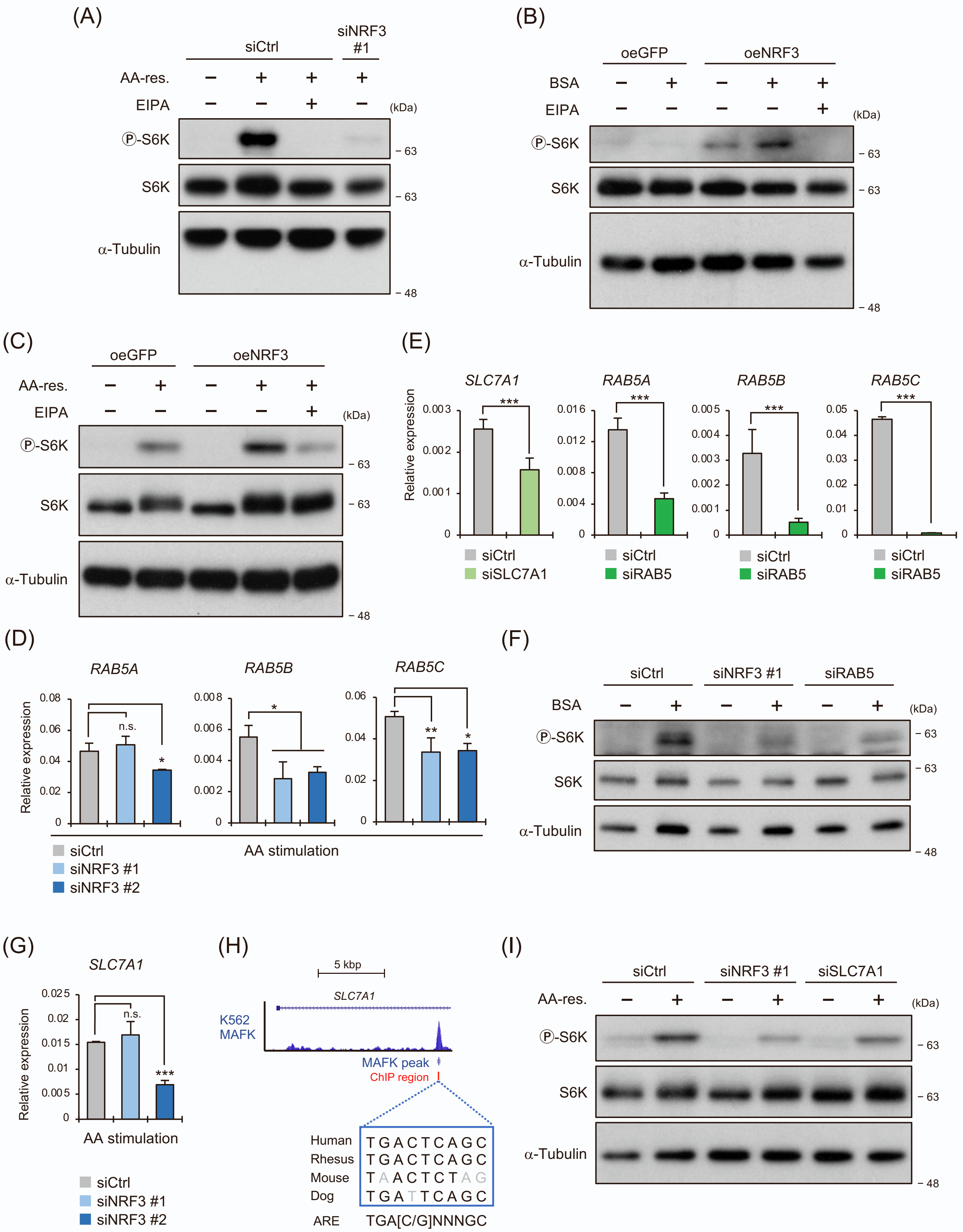

Figure S4. NRF3-mediated induction of macropinocytosis for mTORC1 activation with amino acid stimulation, related to Figure 4

A. The effect of amino acid supplementation and/or macropinocytosis inhibition on mTORC1 activity. HCT116 cells were transfected with the indicated siRNA for two days. Then, the cells were cultured without both FBS and all amino acids for five hours. Finally, these cells were restimulated with or without all amino acids for four hours (+/−AA-res.). As a control, HCT116-siCtrl cells were cultured with 100 μM EIPA during amino acid stimulation (+/−EIPA). ***B and C.*** The effect of NRF3 overexpression on macropinocytosis-mediated mTORC1 activation in response to BSA supplementation (B) or amino acid stimulation (C). In (B), H1299-oeNRF3 or oeGFP cells were cultured without both FBS and all amino acids for five hours. Finally, these cells were stimulated with or without 5% BSA [w/v] for four hours (+/−BSA). In (C), H1299-oeNRF3 or oeGFP cells were cultured as in (A). As a control, H1299-oeNRF3 cells were treated with 75 μM EIPA during amino acid or BSA stimulation (+/−EIPA). ***D.*** The effect of NRF3 knockdown on *RAB5* genes expression in response to amino acid stimulation. HCT116 cells were transfected with the indicated siRNA for two days. The cells were cultured without all amino acids for five hours and then restimulated with all amino acids for 15 min. ***E.*** The knockdown efficiency of siSLC7A1 or siRAB5. HCT116 cells were transfected with the indicated siRNA for two days. siRAB5 means a mixture of siRAB5A, siRAB5B, and siRAB5C. ***F.*** The effect of RAB5s knockdown on mTORC1 activation in response to BSA supplementation. HCT116 cells were transfected with the indicated siRNA and cultured as in Figure 4B. ***G.*** The effect of NRF3 knockdown on *SLC7A1* gene expression in response to amino acid stimulation. HCT116 cells were transfected and cultured as in (D). ***H.*** The genome locus of the *SLC7A1* promoter at the human genome (GRCh37/hg19) shown with ChIP-seq signals of MAFK (blue histogram). Multiple sequences of the candidate ARE in the indicated species were prepared using the web-tool UCSC Genome Browser [60]. The ChIP region is shown as a red rectangle. ***I.*** The effect of SLC7A1 knockdown on mTORC1 activation in response to amino acid stimulation. HCT116 cells were transfected with the indicated siRNA and cultured as in (D). (*E*) Welch *t*-test, (*D, G*) ANOVA followed by Tukey's test: $***p < 0.005$; $**p < 0.01$; $*p < 0.05$; n.s., not significant ($n = 3$, Mean ± SD).

Hirose et al. **Figure S5**

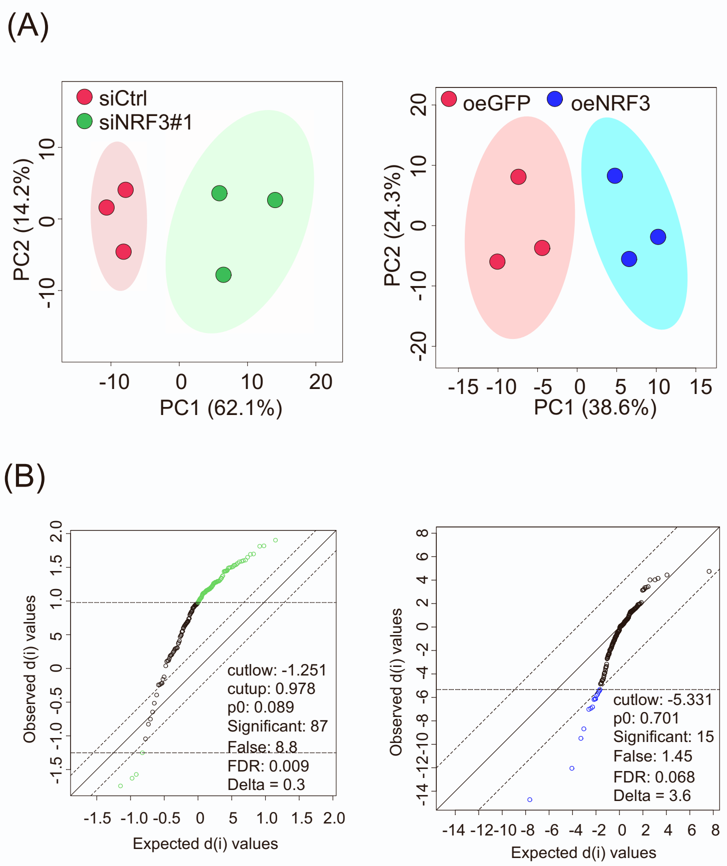

Figure S5. Statistical analysis of our metabolomics data, related to Figure 5

A and B. PCA (A) and significance analysis (B) of metabolomics dataset derived from HCT116-siNRF3 and siCtrl cells with amino acid stimulation or H1299-oeNRF3 and oeGFP cells without amino acid stimulation.

Hirose et al. Figure S6

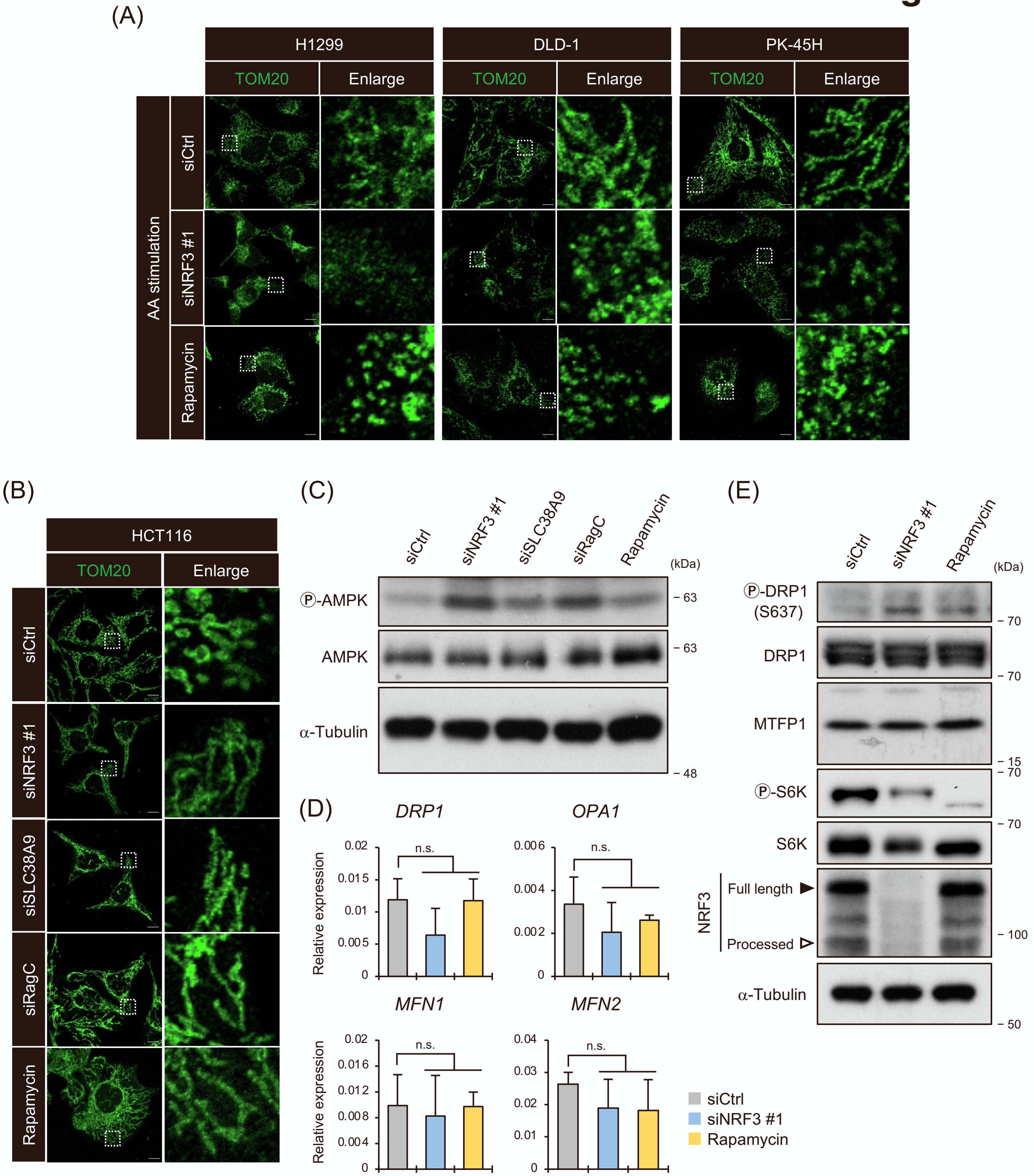

Figure S6. NRF3-mediated mitochondrial quality control with or without amino acid stimulation in various cancer cells, related to Figure 6

A. The effect of indicated gene knockdown or mTORC1 inhibition on mitochondrial morphology in response to amino acid stimulation in various cancer cells. The indicated cells were transfected with indicated siRNA for two days. First, the cells were cultured without all amino acids for 16 h and then restimulated with all amino acids for 15 min. As a control, the siCtrl transfected cells were treated with 10 μM rapamycin for two days and amino acid stimulation. ***B.*** The effect of the indicated gene knockdown or mTORC1 inhibition on mitochondrial morphology without amino acid stimulation. HCT116 cells were transfected with the indicated siRNA for two days. As a control, HCT116-siCtrl cells were treated with 10 μM rapamycin for two days. ***C.*** The effect of indicated gene knockdown or mTORC1 inhibition on p-AMPKα levels without amino acid stimulation. HCT116 cells were transfected and treated as in (B). As a control, HCT116-siCtrl cells were treated with 10 μM rapamycin for two days. ***D.*** The effect of NRF3 knockdown on the mRNA levels of mitochondrial fusion or fission genes without amino acid stimulation. HCT116 cells were transfected and treated as in (B). ANOVA followed by Tukey’s test: n.s., not significant (n = 3, Mean ± SD). ***E.*** The effect of NRF3 knockdown on the protein or phosphorylation levels of the indicated factors without amino acid stimulation. HCT116 cells were transfected and treated as in (B). As a control, HCT116-siCtrl cells were treated with 10 μM rapamycin for two days.

Hirose et al. Figure S7

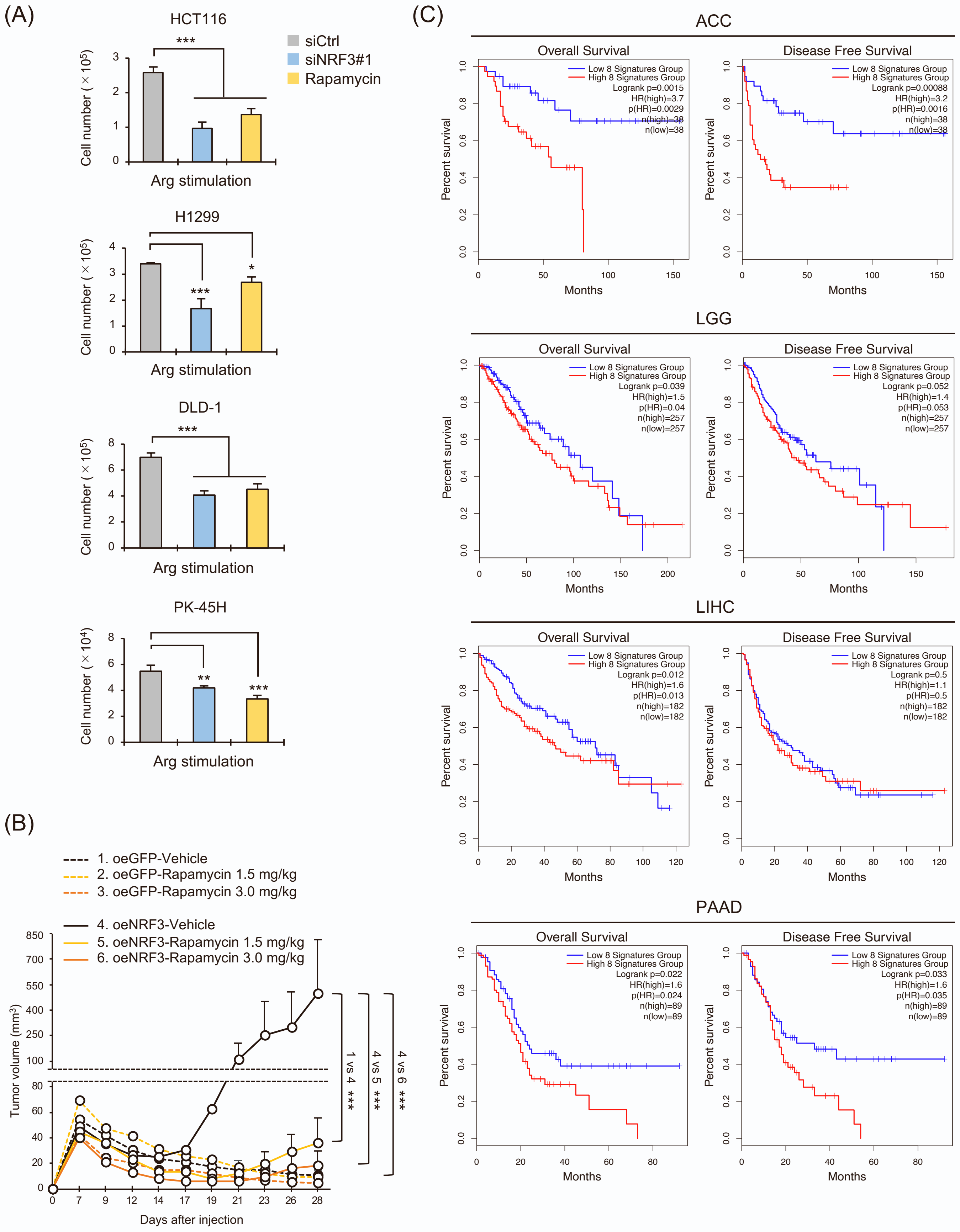

Figure S7. Impact of the NRF3-mTORC1 axis aberration on cancer cell growth and patient prognosis, related to Figure 7

A. The effect of NRF3 knockdown or mTORC1 inhibition on the *in vitro* growth of various cancer cells under arginine stimulation. The indicated cells were transfected with siNRF3 or siCtrl for one day. Then, these cells were cultured without arginine for one day, after which they were restimulated with arginine for 15 min. Cell numbers were finally counted using a hemocytometer. As a control, siCtrl transfected cells were treated with 10 μM rapamycin for two days and arginine stimulation. ANOVA followed by Tukey's test: ***$p < 0.005$; **$p < 0.01$; *$p < 0.05$ ($n = 3$, Mean ± SD). ***B.*** The effect of NRF3 overexpression and rapamycin on tumor growth curves. H1299-oeGFP and H1299-oeNRF3 cells were injected subcutaneously into BALB/cAJcl-Foxn1^{nu} mice, and then, rapamycin (1.5 mg/kg or 3.0 mg/kg) was intraperitoneally administered once every two days. Welch *t*-test: ***$p < 0.005$ ($n = 5$–6, Mean ± SD). ***C.*** Kaplan–Meier plots comparing the overall or disease-free survival of indicated cancer patients with lower or higher expression levels of eight signatures involving *NRF3, RagC, SLC36A1, SLC38A9, RAB5A RAB5B, RAB5C,* and *SLC7A1* genes. ACC, adrenocortical carcinoma; LGG, brain lower-grade glioma, LIHC, liver hepatocellular carcinoma; PAAD, pancreatic adenocarcinoma.
